# Cell type, sub-region, and layer-specific speed representation in the hippocampal–entorhinal circuit

**DOI:** 10.1038/s41598-020-58194-1

**Published:** 2020-01-29

**Authors:** Motosada Iwase, Takuma Kitanishi, Kenji Mizuseki

**Affiliations:** 1 Department of Physiology, Osaka City University Graduate School of Medicine, Osaka, 545-8585 Japan; 2 PRESTO, Japan Science and Technology Agency, Kawaguchi, Saitama, 332-0012 Japan; 3 Center for Molecular and Behavioral Neuroscience, Rutgers University, Newark, NJ 07102 USA

**Keywords:** Hippocampus, Spatial memory, Neural circuits

## Abstract

It has been hypothesised that speed information, encoded by ‘speed cells’, is important for updating spatial representation in the hippocampus and entorhinal cortex to reflect ongoing self-movement during locomotion. However, systematic characterisation of speed representation is still lacking. In this study, we compared the speed representation of distinct cell types across sub-regions/layers in the dorsal hippocampus and medial entorhinal cortex of rats during exploration. Our results indicate that the preferred theta phases of individual neurons are correlated with positive/negative speed modulation and a temporal shift of speed representation in a sub-region/layer and cell type-dependent manner. Most speed cells located in entorhinal cortex layer 2 represented speed prospectively, whereas those in the CA1 and entorhinal cortex layers 3 and 5 represented speed retrospectively. In entorhinal cortex layer 2, putative CA1-projecting pyramidal cells, but not putative dentate gyrus/CA3-projecting stellate cells, represented speed prospectively. Among the hippocampal interneurons, approximately one-third of putative dendrite-targeting (somatostatin-expressing) interneurons, but only a negligible fraction of putative soma-targeting (parvalbumin-expressing) interneurons, showed negative speed modulation. Putative parvalbumin-expressing CA1 interneurons and somatostatin-expressing CA3 interneurons represented speed more retrospectively than parvalbumin-expressing CA3 interneurons. These findings indicate that speed representation in the hippocampal-entorhinal circuit is cell-type, pathway, and theta-phase dependent.

## Introduction

Spatial navigation in humans and animals is an important attribute that enables them to reach their destinations. As part of this process, different brain regions play unique roles in positioning the self in the environment. In particular, ‘place cells’ in the hippocampus^1^ and ‘grid cells’ in the medial entorhinal cortex (MEC)^2^ represent functionally specialised cells that reflect the current location of the animal. To reflect the ongoing self-motion, place cells and grid cells update the associated spatial representation presumably, at least in part, by accessing information regarding the direction and speed of the animal’s current movement.

‘Head-direction cells’ are distributed across multiple brain regions (including the dorsal presubiculum, anterodorsal thalamic nucleus, lateral mammillary nucleus, retrosplenial cortex, entorhinal cortex, and parasubiculum) and encode the direction of the head^3–5^, which is typically the same as the overall direction of ambulation of the animal. Consistent with the concept that head directional information is necessary for generation of grid cell signals^6–8^, disruption of the cell network providing head directional information in the anterior thalamic nuclei would significantly impair both functioning of the grid cells and representation of head direction in the entorhinal cortex and parasubiculum^9^.

In contrast, it has been suggested that speed is encoded by context-invariant, speed-responsive ‘speed cells’ in the MEC^10^. As expected from an internal speedometer, the slope of the firing rate of entorhinal cortical speed cells vs running velocity is preserved across different environments^10^. However, the firing rates, along with the slopes of the regression lines between the speed and the firing rate, of most speed cells in the MEC are lower during dark trials than during light trials^11^. Furthermore, the firing rates of speed cells in the MEC appear to be modulated by a combination of locomotion, optic flow, and landmark, with optic flow being more influential when it is faster than expected^12^. Therefore, the firing rates of the speed cells in the MEC can be determined by both internal self-motion cues and visual information.

It has been suggested that running speed and head direction are integrated across short time windows to obtain instantaneous displacement information, which is used to update the representation of the animal’s location^6–8,13–18^ (also see Dannenberg *et al*.^19^). The MEC comprises grid cells, head-direction cells, and speed cells, and is assumed to perform the path integration^8,10,20–23^. Consistent with this notion, the firing rate of grid cells has been shown to be modulated by speed^20,24^. In addition, passive transport abolishes both the velocity modulation of theta oscillations and grid cell firing patterns^25^. Furthermore, spatially modulated cells in the superficial layers of the MEC show anticipatory firing field shifts between high and low speeds, and between positive and negative accelerations that increase with the absolute acceleration threshold^10^. This observation is consistent with the notion that the activity of spatially modulated cells in the superficial MEC is driven by speed cells with prospective coding^10^. The information encoded by speed cells may thus be utilised to optimise coding in the MEC because the neurons therein vary their coding properties dynamically in such a way that they become more informative about position, head direction, and the conjunction of position and head direction when an animal is running at higher speeds^26^.

The firing rate of the hippocampal place cell has also been shown to correlate with the speed at which rats traverse the place field^27–34^. In the CA1 region, both firing rate gains by the running speed^32^ and spatial information encoded by pyramidal cells^32,35,36^ increase from the temporal to septal direction. This is in line with the idea that speed information is utilised by the hippocampus to compute the current position. Only a low correlation has been observed between the speed and firing rates of MEC grid cells and head-direction cells inside their fields, whereas a higher correlation has been observed between the speed and firing rates of hippocampal place cells inside their fields^10^. These findings suggest a stronger link between speed and space coding in the hippocampus^10^.

The power and frequency of theta oscillations in the entorhinal cortex and hippocampus both increase with an increase in running speed^37–41^. Furthermore, a stronger positive correlation has been demonstrated between running speed and theta power in the CA1 region than between running speed and theta power in the dentate gyrus (DG) region^39^. Hippocampal CA1 gamma power and frequency also increase as a function of running speed^42–44^. Accordingly, the stratum lacunosum-moleculare of the CA1 region has been shown to exhibit a strong positive correlation between theta-gamma coupling and running speed^45^, suggesting that the speed signal of theta-modulated gamma inputs are conveyed from the entorhinal cortex to the hippocampus^46,47^. Consistent with this notion, the majority of speed cells in the MEC are suggested to encode speed prospectively, whereas those in the hippocampus are suggested to encode speed retrospectively^10^. Therefore, entorhinal fast-spiking speed cells that project to the hippocampus^48^ may play an important role in this overall process.

Since the first report by McNaughton *et al*., in 1983^27^, the velocity modulation of neuronal firing in the hippocampus has been extensively examined^20,24,27–34,49–53^. After the recent discovery of speed cells in the MEC^10^, speed representation in the hippocampus-entorhinal system has received considerable attention^11,48,54–56^. However, a systematic and quantitative comparison of speed representation in various cell types in the associated sub-regions and layers of the hippocampal–entorhinal circuit remains to be achieved. By analysing the simultaneously recorded neuronal activities of the dorsal hippocampus and MEC during the exploration of an open field^57–59^, we compared the speed representation in distinct cell types across sub-regions and layers of the hippocampal–entorhinal circuit. Our findings indicate that distinct cell types at each anatomical station in the hippocampal–entorhinal circuit represent speed information differently.

## Results

### Proportion of speed cells

**Figure 1:**
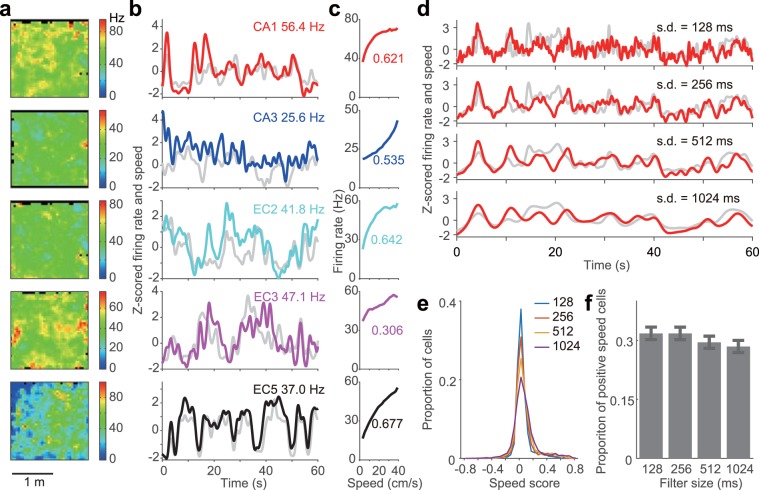
Example of positive speed (p-Speed) cells and the effect of Gaussian filter size on estimating speed modulation. (**a–c**) Five p-Speed cells recorded from CA1, CA3, medial entorhinal cortex layer 2 (EC2), layer 3 (EC3), and layer 5 (EC5) in an open field (180 × 180 cm). For each cell, the firing-rate map (**a**; pixel size, 5 cm × 5 cm), Z-scored firing rate (colour) and running speed (grey) (**b**) and mean firing rate as a function of running speed (**c**) (bin size: 2 cm/s) are shown. Numbers (Hz) in (**b**) represent the mean firing rates when animals were running at 2–50 cm per second. Numbers in (**c**) represent speed scores. (**d–f**) Correlation between instantaneous firing rate and running speed using different sizes of Gaussian filters. (**d**) Z-scored firing rate (red) and running speed (grey) of a representative p-Speed cell in the open field. Different size of Gaussian filter (SDs, 128, 256, 512 and 1024 ms) were used for smoothing both the firing rate and running speed. (**e**) Distribution of speed scores using different Gaussian filter sizes. Cells in the hippocampus (CA1 and CA3) and entorhinal cortex (EC2, EC3, and EC5) were combined. Note that the larger the size of the filter the larger the absolute value of the speed scores. (**f**) Proportion of p-Speed cells using different sizes of Gaussian filters ( ± 95% Clopper–Pearson confidence intervals). A Gaussian filter with a SD of 512 ms was used for the rest of the study. However, qualitatively similar results were observed using filters differing in sizes (SDs, 128, 256, 512, and 1024 ms). SD, standard deviation.

To comprehensively understand the neuronal correlates of locomotion speed in the hippocampus and entorhinal cortex, we analysed the activity of single units in the CA1 and CA3 regions of the dorsal hippocampus (n = 2,042 cells) and the MEC (n = 1,313 cells) in freely moving rats (n = 4) exploring a square open-field arena. The rats exhibited a wide range of instantaneous speeds, typically from 0 to 50 cm/s (n = 60 sessions; arena size, 180 cm × 180 cm or 120 cm × 120 cm)^57,59^. As widely reported^10–12,20,24,27–34,43,49–52,56,60^, some neurons in the hippocampus and entorhinal cortex increased their firing rates as a function of running speed (Fig. 1). Given that the effect of running speed on the activities of distinct classes of entorhinal cortical cells, such as grid cells, border cells, and head-direction cells have been extensively studied^10–12,20,24–26^, in this study, we focused on the layer specificity and the differences between principal neurons and interneurons, and pyramidal cells and stellate cells in the entorhinal cortex. To compare the correlation between firing rate and running speed in distinct cell types, recorded neurons were first classified as either principal neurons or interneurons based on spike waveforms and the physiological criteria outlined in previous studies^57,58,61–64^ (see Methods section). In total, we analysed 1,545 CA1 cells (1,266 pyramidal cells, 183 interneurons, 96 unclassified cells), 497 CA3 cells (378 pyramidal cells, 99 interneurons, 20 unclassified cells), 352 cells (287 principal neurons, 51 interneurons, 14 unclassified cells) in MEC layer 2 (EC2), 468 cells (311 principal neurons, 113 interneurons, 44 unclassified cells) in MEC layer 3 (EC3), and 493 cells (357 principal neurons, 51 interneurons, 85 unclassified cells) in MEC layer 5 (EC5; Supplementary Table S1).

**Figure 2:**
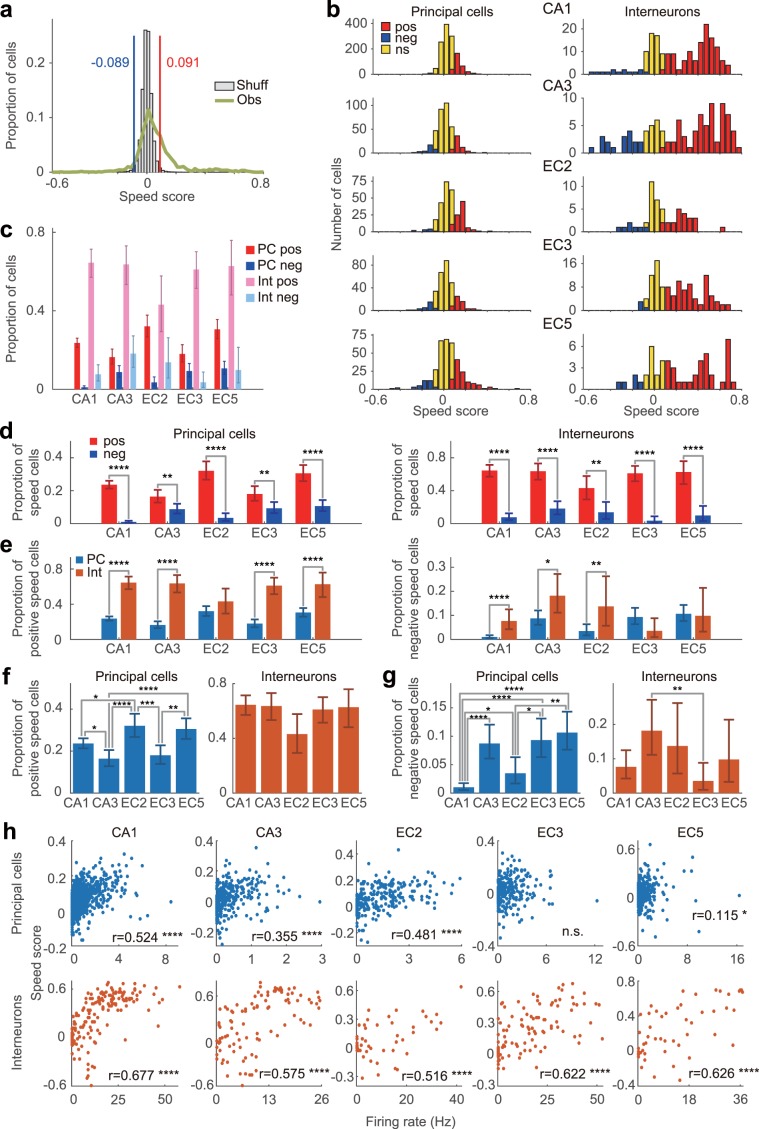
Distribution of speed scores and proportions of speed cells. (**a**) Distributions of observed speed scores (‘Obs’) and speed scores obtained by 100 times shuffles per cell (‘Shuff’, normalised by number of shuffles). All cells recorded from the hippocampus and medial entorhinal cortex were combined. The blue and red lines indicate the 1st and 99th percentiles of the speed scores obtained from shuffling, respectively (values at the top). (**b**) Distributions of speed scores of principal neurons (left) and interneurons (right) in each sub-region/layer. Pos, p-Speed cells. Neg, n-Speed cells. Ns, non-speed cells. (**c**) Proportions of p-Speed and n-Speed cells for each sub-region/layer ( ± 95% Clopper–Pearson confidence intervals). PC pos, p-Speed principal cells; PC neg, n-Speed principal cells; Int pos, p-Speed interneurons; Int neg, n-Speed interneurons. (**d–g**) Same data as (**c**) but displayed in different ways to facilitate comparison between cell types, sub-regions/layers, and positive vs negative speed cells. (**d**) Proportion of p-Speed (pos, red) and n-Speed (neg, blue) principal cells (left) and interneurons (right) in CA1, CA3, EC2, EC3, and EC5 ( ± 95% Clopper–Pearson confidence intervals). (**e**) Proportion of p-Speed (left) and n-Speed (right) principal cells (PC, light blue) and interneurons (Int, orange) in CA1, CA3, EC2, EC3, and EC5 ( ± 95% Clopper–Pearson confidence intervals). (**f**) Proportion of p-Speed principal cells (left) and interneurons (right) in CA1, CA3, EC2, EC3, and EC5 ( ± 95% Clopper–Pearson confidence intervals). (**g**) Proportion of n-Speed principal cells (left) and interneurons (right) in CA1, CA3, EC2, EC3, and EC5 ( ± 95% Clopper–Pearson confidence intervals). Fisher’s exact test with Bonferroni correction, *P < 0.05, **P < 0.01, ***P < 0.001, ****P < 0.0001. (**h**) Relationship between speed score and firing rate of principal neurons (top) and interneurons (bottom) in CA1, CA3, EC2, EC3, and EC5. r, correlation coefficient. *P < 0.05, ****P < 0.0001, n.s. (not significant).

We followed the guidelines provided by Kropff *et al*.^10^, and for each cell, we calculated a speed score, which is defined as the Pearson product-moment correlation coefficient between the animal’s running speed and the instantaneous firing rate of the cell (Figs. 1d–f and 2a). To this end, we used periods with a running speed of > 2 cm/s to filter out the static periods^10^ (see Methods section). Cells with speed scores greater than the 99th percentile of a shuffled data distribution of all hippocampal and entorhinal cortical cells analysed were classified as ‘p-Speed cells (positive speed cells)’ (Fig. 2a; speed score threshold, 0.091) whereas cells with speed scores lower than the first percentile of the shuffled distribution were classified as ‘n-Speed cells (negative speed cells)’ (Fig. 2a; speed score threshold, −0.089)^10^. Accordingly, hereafter, principal neurons and interneurons with a significant positive (or negative) speed modulation are referred to as ‘p-Speed (or n-Speed) principal neurons’ and ‘p-Speed (or n-Speed) interneurons’, respectively. The numbers and fractions of each type of speed cell in each sub-region are summarised in Supplementary Table S1. We acknowledge that neurons classified as ‘speed cells’ based on the aforementioned criteria can encode other behavioural correlates^26,55,65^ (e.g., space, head direction; Supplementary Figs. 1–5). Furthermore, a spurious correlation between speed and the firing rate may be caused if neurons code a particular place field by emitting a particular number of spikes, independent of the animal’s speed through the place field^33^. A recent modelling study suggests that variability in the initial theta phase at the entry to the place field, especially at a fast running speed, could cause a spurious correlation between speed and the firing rate, even when there is no correlation between the speed and firing rate in the model^66^. Nonetheless, the nomenclature ‘speed cells’, instead of a lengthy description of ‘cells whose firing rates are statistically significantly correlated with running speed’, is being used for the sake of convenience^55^.

Consistent with previous reports^10,55,67^, proportions of p-Speed cells were higher than those of n-Speed cells in both the principal neurons and interneurons of the hippocampus and MEC (Fig. 2b–d). Previous reports have suggested that most speed cells in the hippocampal CA1 region and MEC are fast-spiking interneurons^10,11,48,55^. Consistent with previous reports, in the CA1, CA3, EC3, and EC5 regions, a larger proportion of interneurons were found to be p-Speed cells than principal neurons (Fig. 2e; Fisher’s exact test, P < 0.0001). In contrast, the proportions of p-Speed cells in principal neurons and interneurons in EC2 were not significantly different (Fig. 2e). Conversely, proportions of n-Speed cells were higher in interneurons than in principal neurons in CA1, CA3, and EC2 (Fig. 2e). The proportion of p-Speed cells in principal neurons was significantly larger in EC2 than in CA1, CA3, and EC3 (Fig. 2f). The proportion of p-Speed cells in the principal neurons of CA3 was significantly smaller than those in CA1, EC2, and EC5 (Fig. 2f). In view of a report showing that a fraction of CA2 pyramidal neurons exhibited negative speed modulation and code for space during immobility^68^, we compared the proportion of n-Speed cells across sub-regions/layers. Our results indicated that the proportion of n-Speed cells in principal neurons was lowest in the CA1 region, whereas that in interneurons was significantly higher in CA3 than in EC3 (Fig. 2g). In summary, the principal neurons and interneurons present in distinct sub-regions/layers contain differing proportions of p-Speed and n-Speed cells.

It has been reported that there is no significant correlation between mean firing rates and speed scores^10,48^ (but see Perez-Escobar *et al*.^11^ and Gois & Tort^55^). However, we found a significant correlation between the mean firing rates during walking and the speed scores of principal neurons and interneurons (Fig. 2h). Furthermore, a significant correlation was observed between the mean firing rates during walking and the absolute speed score values for principal neurons (CA1, r = 0.542; CA3, r = 0.239; EC2, r = 0.410; EC3, r = 0.193; EC5, r = 0.369; all P < 0.0001) and interneurons (CA1, r = 0.737; CA3, r = 0.572; EC2, r = 0.631; EC3, r = 0.619; EC5, r = 0.668; all P < 0.0001). These findings indicated that the activity of neurons with a higher firing rate is more strongly correlated with speed.

### Preferred theta phase of p-speed and n-speed cells

**Figure 3:**
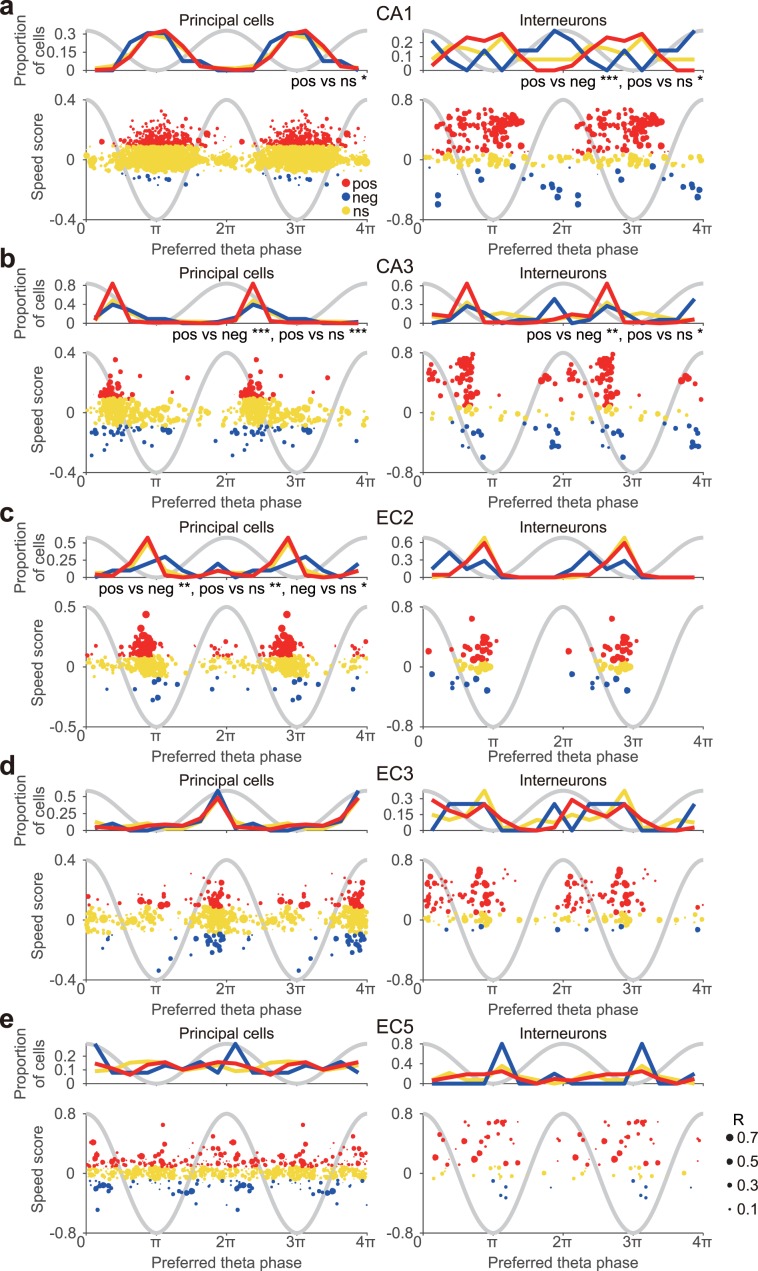
Relationship between the preferred theta phase and speed score of individual neurons in the hippocampus and entorhinal cortex. The distribution of the preferred theta phase (top) and the relationship between the speed score and preferred theta phase (bottom) of individual p-Speed (pos, red), n-Speed (neg, blue) and non-speed (ns, yellow) principal neurons (left) and interneurons (right) in the hippocampal CA1 (**a**) and CA3 (**b**) areas, EC2 (**c**), EC3 (**d**), and EC5 (**e**). Sinusoidal grey lines, idealised theta oscillations recorded from EC3. The size of each circle represents the strength of theta locking (mean resultant length, *R*) of each neuron. Watson U^2^ test, *P < 0.05, **P < 0.01, ***P < 0.001.

When an animal is walking, theta oscillations are prominent in the hippocampus and entorhinal cortex^69,70^. By optogenetically controlling hippocampal theta oscillations, it has recently been shown that the hippocampal theta oscillations regulate the speed of locomotion in mice^71^. As different cell types in these brain regions preferentially fire at a distinct theta phase during theta oscillations^58,69^, we examined the relationship between the preferred theta phase and the speed representation of individual neurons (Fig. 3). Given the availability of an electrode in EC3 for all recording sessions, we used EC3 theta oscillations, which are highly coherent and in phase with those of the CA1 pyramidal layer, as a common reference for comparing the spike timing across sub-regions/layers^58^. Our results indicated that p-Speed and n-Speed hippocampal interneurons fired at distinct theta phases. In CA1, p-Speed interneurons fired preferentially at the trough of the theta oscillations while n-Speed interneurons preferred the peak (Fig. 3a). Similarly, in CA3, p-Speed interneurons preferentially fired during the descending theta phase, while approximately half of n-Speed interneurons preferred the peak (Fig. 3b). Moreover, principal neurons in CA3 and EC2 exhibited a significant relationship between preferred theta phase and positive vs negative speed modulation (Fig. 3b,c). Overall, in hippocampal CA1 and CA3, p-Speed interneurons preferred the descending/trough whereas n-Speed interneurons preferred the peak of theta oscillations.

### Distribution of speed scores, speed slope, and speed information in p-speed cells

**Figure 4:**
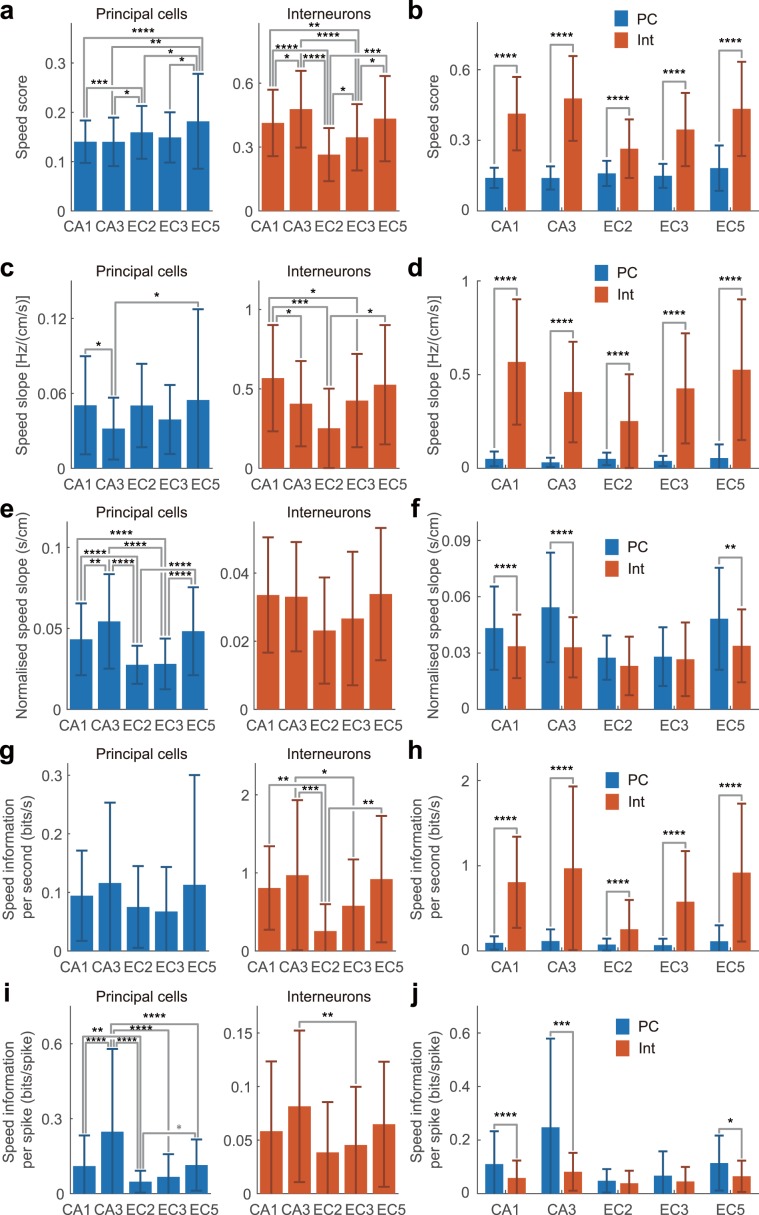
Comparison of speed score, speed slope, and speed information of p-Speed cells. (**a**,**c**,**e**,**g**,**i**) Speed score (**a**), speed slope [Hz/(cm/s)] (**c**), normalised speed slope (s/cm) (**e**), speed information per second (bits/s) (**g**), and speed information per spike (bits/spike) (**i**) of p-Speed principal neurons (left) and interneurons (right) in each region/layer (mean ± SD). Analysis of variance (ANOVA) followed by Bonferroni test, *P < 0.05, **P < 0.01, ***P < 0.001, ****P < 0.0001. (**b**,**d**,**f**,**h**,**j**) Same data as (**a**,**c**,**e**,**g**,**i**) but displayed in a different way to facilitate comparison between principal neurons and interneurons. Speed score (**b**), speed slope [Hz/(cm/s)] (**d**), normalised speed slope (s/cm) (**f**), speed information per second (bits/s) (**h**), and speed information per spike (bits/spike) (**j**) of p-Speed principal cells (PC, light blue) and interneurons (Int, orange) in each region/layer (mean ± SD). *t*-test, *P < 0.05, **P < 0.01, ***P < 0.001, ****P < 0.0001.

Next, we compared speed scores across sub-regions/layers and between cell types. We confined our analysis to p-Speed cells because the numbers of n-Speed cells were too small to perform a comprehensive comparison across regions/layers and cell types. Within the p-Speed principal neurons in the different sub-regions/layers, the speed scores were the highest in EC5 (Fig. 4a, with means and standard deviations [SDs] summarised in Supplementary Table S2). In contrast, within the p-Speed interneurons in the different sub-regions/layers, the speed scores were the lowest in EC2 (Fig. 4a). It has been previously demonstrated that interneurons have higher speed scores than pyramidal cells in the hippocampal CA1 area^55^. Our results were consistent with this finding and further showed that the speed scores of p-Speed interneurons were consistently higher than those of p-Speed principal neurons in all regions and layers (Fig. 4b).

Speed scores represent the strength of the correlation between instantaneous locomotion speed and firing rates; however, they do not provide information on how the locomotion speed is represented by the firing rate. To compare the firing rate gain by speed, we calculated the slope of the regression line between the running speed and instantaneous firing rate of the p-Speed cells. Within the p-Speed principal neurons, CA3 p-Speed principal neurons displayed shallower speed slopes than those located in CA1 and EC5 (Fig. 4c; means and SDs summarised in Supplementary Table S3). Within the p-Speed interneurons, EC2 p-Speed interneurons displayed shallower speed slopes than those located in CA1 and EC5 (Fig. 4c). Furthermore, the speed slopes of p-Speed interneurons were observed to be steeper than those of p-Speed principal neurons in both the hippocampus and entorhinal cortex (Fig. 4d). Significant correlations between the speed score and speed slope in p-Speed principal neurons (CA1, r = 0.674; CA3, r = 0.551; EC2, r = 0.564; EC3, r = 0.564; EC5, r = 0.669; all P < 0.0001) and interneurons (CA1, r = 0.745; CA3, r = 0.786; EC2, r = 0.836; EC3, r = 0.733; EC5, r = 0.853; all P < 0.0001) were observed. The distribution of firing rates of individual neurons is skewed and spans at least three orders of magnitude in the hippocampus and entorhinal cortex^61^. Moreover, individual neurons can represent information by the relative but not absolute changes in firing rate from the baseline. Therefore, we also compared the normalised speed slope, which was defined as the slope of the regression line between the running speed and instantaneous firing rate divided by the mean firing rate. p-Speed principal neurons in EC2 and EC3 demonstrated shallower normalised speed slopes than those located in CA1, CA3, and EC5 (P < 0.01, analysis of variance [ANOVA] followed by the Bonferroni test, P < 0.0001, Fig. 4e). There was no significant difference between layers/regions in terms of the normalised speed slope of p-Speed interneurons (Fig. 4e). The p-Speed principal neurons exhibited steeper normalised speed slopes than p-Speed interneurons in CA1, CA3, and EC5 (Fig. 4f). Furthermore, significant correlations were observed between the speed scores and normalised speed slopes in p-Speed principal neurons (CA1, r = 0.124, P < 0.05; CA3, r = 0.455, P < 0.001; EC2, r = 0.581, P < 0.0001; EC3, r = 0.537, P < 0.0001; EC5, r = 0.377, P < 0.0001) and CA3 interneurons (r = 0.548, P < 0.0001).

To further quantify the speed representation by individual neurons, we calculated the ‘speed information per second’ (bits per second), which was defined according to the definition of spatial information^72^ (see Methods section). Within p-Speed principal neurons, there were no significant differences between sub-regions/layers in terms of speed information per second (Fig. 4g). p-Speed interneurons in EC2 had less speed information per second than did p-Speed interneurons in CA1, CA3, and EC5 (Fig. 4g; means and SDs summarised in Supplementary Table S4). When we compared p-Speed principal neurons and interneurons, p-Speed interneurons had higher speed information per second (Fig. 4h). Since higher firing-rate neurons tend to have higher speed information rate (bits per second), we also compared ‘speed information per spike’ (bits per spike). Among the p-Speed principal neurons, p-Speed principal neurons in CA3 showed the highest speed information per spike (Fig. 4i). The p-Speed principal neurons in EC2 demonstrated lower speed information per spike than those located in CA1, CA3, and EC5 (Fig. 4i). When we compared p-Speed principal neurons and interneurons, overall, p-Speed principal neurons had higher speed information per spike than p-Speed interneurons in CA1, CA3, and EC5 (Fig. 4j). In summary, compared to p-Speed cells in CA1, CA3, and EC5, p-Speed cells in EC2 and EC3, a major input to the hippocampus, had similar or smaller speed slopes, normalised speed slopes, speed information per second, and speed information per spike.

### Temporal shifts of p-speed cells

**Figure 5:**
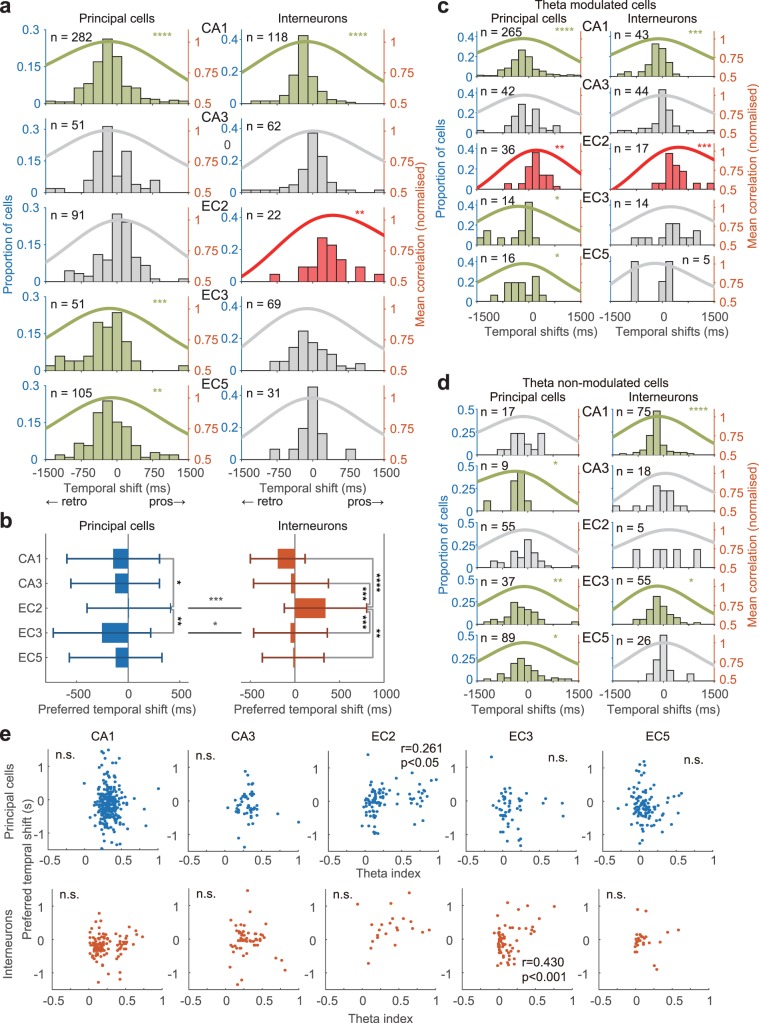
Prospective and retrospective speed representation of p-Speed cells. (**a**) Bars represent preferred temporal shift distribution of individual neurons, which maximise the correlation between instantaneous firing rate and speed. Lines represent mean normalised correlation. Light green, significantly retrospective representation. Magenta, significantly prospective representation. *t*-test, **P < 0.01, ***P < 0.001, ****P < 0.0001. (**b**) Preferred temporal shifts of individual neurons (mean ± SD). ANOVA followed by Bonferroni test, *P < 0.05, **P < 0.01, ***P < 0.001, ****P < 0.0001. (**c,d**) The same data as (**a**), but theta-modulated cells (**c**) and theta non-modulated cells (**d**) are shown separately. A theta index of 0.2 was used to divide cells into theta-modulated and non-modulated cells (see Methods section). Light green, significantly retrospective representation. Magenta, significantly prospective representation. *t*-test, *P < 0.05, **P < 0.01, ***P < 0.001, ****P < 0.0001. (**e**) Relationship between preferred temporal shift and theta index of p-Speed cells. Principal neurons, top. Interneurons, bottom. r, Pearson product-moment correlation coefficient. n.s. (not significant).

It has been reported that the majority of p-Speed cells in the hippocampus encode speed retrospectively whereas those in the MEC encode speed prospectively^10,55^. However, there has been no comprehensive comparison of temporal shifts of speed representation between sub-regions (e.g. CA1 vs CA3) or across MEC layers and cell types (principal neurons vs interneurons). Therefore, we examined the temporal biases of p-Speed cells in distinct cell types across sub-regions and layers. To this end, we calculated Pearson product-moment correlation coefficients between running speeds and instantaneous firing rates that were shifted in a step of 25.6 ms at the interval between −1,536 ms and 1,536 ms. The shift that maximised the correlation coefficient was termed the ‘preferred temporal shift’ of that neuron. Our results indicated that most p-Speed principal neurons and interneurons in CA1 and p-Speed principal neurons in EC3 and EC5 represented speed retrospectively, whereas most p-Speed interneurons in EC2 represented speed prospectively (Fig. 5a; means and SDs summarised in Supplementary Table S5). As a population, p-Speed cells in CA3, p-Speed principal neurons in EC2, and p-Speed interneurons in EC3 and EC5 did not display any significant bias towards either prospective or retrospective representation (Fig. 5a). The preferred temporal shifts of p-Speed principal neurons in CA1 and EC3 were found to be significantly biased towards retrospective representation when compared with p-Speed principal neurons in EC2 (Fig. 5b; CA1, –145 ms vs EC2, 7 ms, P < 0.05; EC3, –254 ms vs EC2, 7 ms, P < 0.01, ANOVA followed by Bonferroni test). Similarly, the preferred temporal shift of p-Speed interneurons in EC2 was significantly biased towards prospective representation when compared with p-Speed interneurons in CA1, CA3, EC3, and EC5 (Fig. 5b; EC2, 343 ms; vs CA1, –193 ms, P < 0.0001; vs CA3, –44 ms, P < 0.001; vs EC3–50 ms, P < 0.001; vs EC5, –20 ms, P < 0.01). The p-Speed EC2 interneurons represented speed significantly more prospectively than did the p-Speed EC2 principal neurons (Fig. 5b; interneurons, 343 ms vs principal neurons, 7 ms, P < 0.001). Similarly, p-Speed principal neurons in EC3 represented speed significantly more retrospectively than p-Speed interneurons in EC3 (Fig. 5b; principal neurons, –254 ms vs interneurons, –50 ms, P < 0.05). It should be noted that the mean preferred temporal shift of p-Speed interneurons in EC2 (343 ms) was much longer than the previously reported preferred temporal shift in the MEC (54–82 ms)^10^, likely because the previous study did not analyse interneurons and principal neurons separately in the different layers of MEC^10^.

It has been previously reported that only theta-modulated cells encode speed prospectively in the MEC;^10^ therefore, we next analysed the temporal shift of theta-modulated and non-modulated p-Speed cells separately (Fig. 5c,d; see Methods section). Consistent with previous reports^10^, we found that the majority of theta-modulated p-Speed principal neurons and interneurons in EC2 represented speed prospectively (Fig. 5c). However, theta-modulated cells in CA1, CA3, EC3, and EC5 did not show a significant temporal bias towards prospective representation (Fig. 5c). Furthermore, we found that the theta index and preferred temporal shift exhibited a significant correlation in principal neurons in EC2 (r = 0.261, P < 0.05) and interneurons in EC3 (r = 0.43, P < 0.001), but not in cells located in CA1, CA3, or EC5, interneurons in EC2, or principal neurons in EC3 (Fig. 5e). It has been suggested that theta-modulated interneurons, relative to non-modulated interneurons, have steeper slopes of running speed vs normalised firing rate in CA1^49^. Therefore, we examined the relationship between theta index and normalised speed slope. We found a significant correlation between theta index and normalised speed slope in p-Speed CA1 pyramidal cells (r = 0.263, P < 0.0001) but not in p-Speed CA1 interneurons or in p-Speed principal neurons and interneurons in CA3, EC2, EC3, and EC5.

Collectively, our results indicate that each sub-region/layer has a different degree of prospective vs retrospective speed representation. Theta modulation and temporal shifts significantly correlated only in the neurons present in the superficial MEC. Even within the same sub-region/layer, p-Speed principal neurons and interneurons appear to have distinct prospective/retrospective speed representation.

### Preferred theta phase vs temporal shifts in p-Speed cells

**Figure 6:**
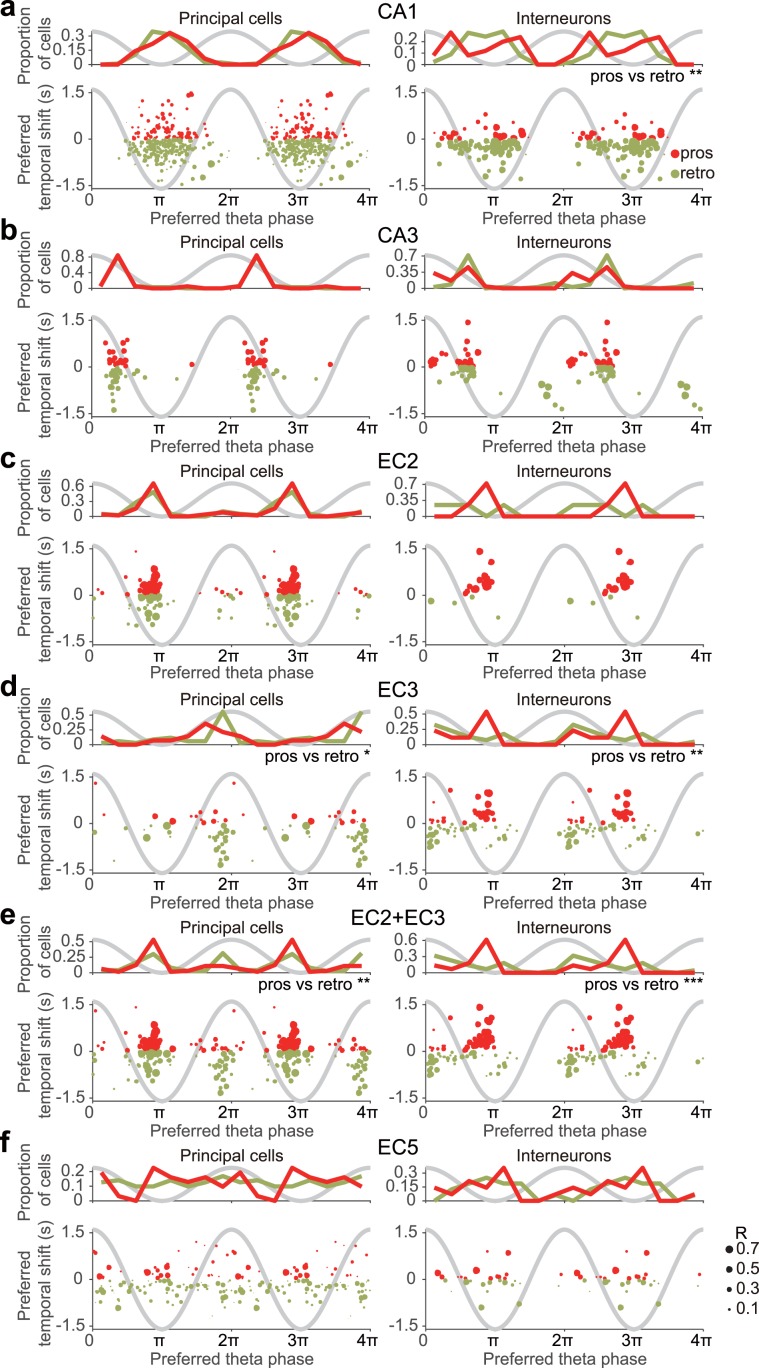
Relationship between the preferred temporal shift and preferred theta phase of individual p-Speed cells in the hippocampus and entorhinal cortex. Distribution of the preferred theta phase (top) and the relationship between the preferred temporal shift and preferred theta phase (bottom) of individual prospective (pros, red) and retrospective (retro, light green) p-Speed principal neurons (left) and interneurons (right) in CA1 (**a**), CA3 (**b**), EC2 (**c**), EC3 (**d**), EC2 + EC3 (**e**), and EC5 (**f**). Sinusoidal grey lines, idealised theta oscillations recorded from EC3. Watson U^2^ test, *P < 0.05, **P < 0.01, ***P < 0.001. The size of each circle represents the strength of theta locking (mean resultant length, *R*) of each neuron.

To better understand the potential relationship between theta oscillations and retrospective/prospective speed representation, we next examined the relationship between the preferred theta phase and the preferred temporal shift of individual p-Speed cells, using EC3 theta oscillations as a common reference^58^ (Fig. 6). In p-Speed principal neurons, there was little correlation between the preferred theta phase and preferred temporal shifts (Fig. 6, left column). However, there was a noticeable correlation between the preferred theta phase and preferred temporal shifts in CA1 and EC3 p-Speed interneurons (Fig. 6a,d). Prospective p-Speed CA1 interneurons preferentially fired at the descending and ascending phases of theta oscillations, whereas retrospective p-Speed CA1 interneurons preferred the trough (Fig. 6a). Prospective p-Speed EC3 interneurons preferentially fired at the trough of theta oscillations, whereas retrospective p-Speed EC3 interneurons preferred the peak (Fig. 6d). EC2 p-Speed interneurons displayed prospective representation and fired near the trough (Fig. 6c). As such, in the superficial layer of MEC, p-Speed EC2 principal neurons and prospective p-Speed EC2 and EC3 interneurons preferentially fired near the trough, whereas retrospective p-Speed EC3 principal neurons and retrospective p-Speed EC3 interneurons preferentially fired at the peak of theta (Fig. 6c–e). In summary, in CA1 and EC3, retrospective and prospective p-Speed interneurons prefer distinct theta phases.

### Speed representation in EC2 pyramidal and stellate cells

**Figure 7:**
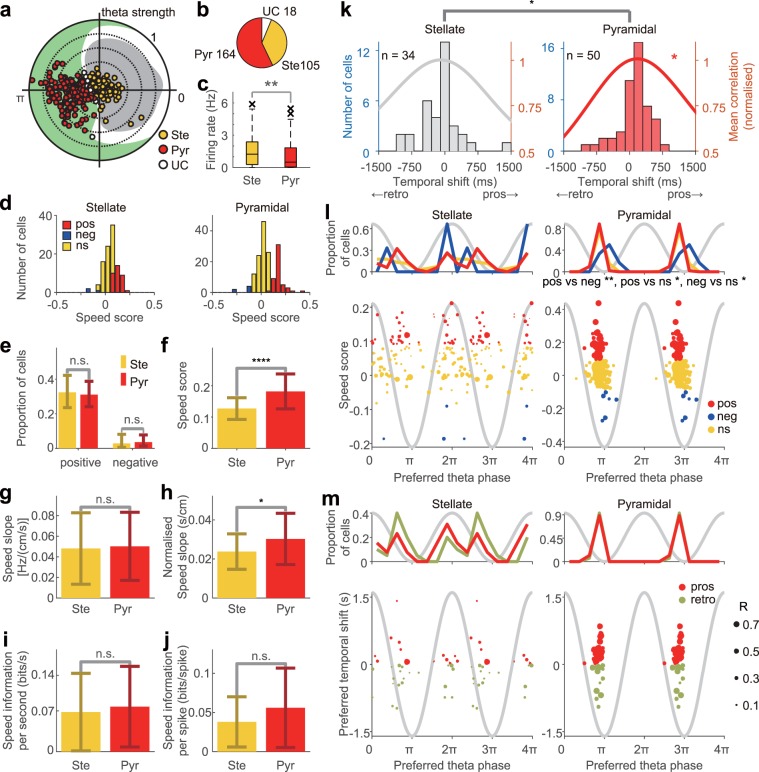
Speed representation of putative stellate and pyramidal cells in layer 2 of the medial entorhinal cortex (EC2). (**a**) The polar plot of preferred theta phase and magnitude of theta locking (mean resultant length) of EC2 principal neurons. Cells were classified as putative stellate cells (Ste, yellow) or pyramidal cells (Pyr, red) using the previously published criteria^77^. UC, unclassified cells. Theta oscillations recorded in EC2 were used as a reference. (**b**) The proportion of stellate (Ste), pyramidal (Pyr) and unclassified (UC) cells. The numbers indicate the numbers of each cell type. (**c**) Box plots of firing rate of stellate (Ste) and pyramidal (Pyr) cells. Wilcoxon rank sum test, **P < 0.01. (**d**) Distribution of speed scores of stellate (left) and pyramidal cells (right). Pos, p-Speed cells. Neg, n-Speed cells. Ns, non-speed cells. (**e**) Proportion of p-Speed (positive) and n-Speed (negative) cells in stellate (Ste) and pyramidal (Pyr) cells ( ± 95% Clopper–Pearson confidence intervals). (**f–j**) Comparison of speed score (**f**), speed slope [Hz/(cm/s)] (**g**), normalised speed slope (s/cm) (**h**), speed information per second (bits/s) (**i**), and speed information per spike (bits/spike) (**j**) of p-Speed stellate (Ste) and pyramidal (Pyr) cells (mean ± SD). *t*-test, ****P < 0.0001, *P < 0.05, n.s. (not significant). (**k**) Distribution of preferred temporal shifts (bars) and mean normalised correlation (lines) of p-Speed stellate (left) and pyramidal (right) cells. Pyramidal cells showed significant prospective representation. *t*-test, * P < 0.05. Preferred temporal shifts were significantly different between stellate and pyramidal cells. *t*-test, * P < 0.05. (**l**) The distribution of the preferred theta phase (top) and the relationship between the speed score and preferred theta phase (bottom) of individual p-Speed (pos, red), n-Speed (neg, blue), non-speed (ns, yellow) stellate (left) and pyramidal cells (right). (**m**) The distribution of the preferred theta phase (top) and the relationship between the preferred temporal shift and preferred theta phase (bottom) of individual prospective (pros, red) and retrospective (retro, light green) p-Speed putative stellate (left) and pyramidal cells (right). Sinusoidal grey lines in (**l**,**m**), idealised theta oscillations recorded from EC3. Size of each circle represents the strength of the theta locking (mean resultant length, *R*) of each neuron. Watson U^2^ test, * P < 0.05, ** P < 0.01.

The two main EC2 principal neuron cell types are pyramidal and stellate cells^73^. It has recently been suggested that stellate cells project to the DG, CA2, and CA3 regions, whereas pyramidal cells project to CA1 and mostly innervate interneurons^74–76^. To understand how speed information is transferred from EC2 to DG, CA3, and CA1, we classified EC2 principal neurons as either stellate or pyramidal cells using previously proposed physiological criteria^77^ (Fig. 7a; see Methods section) and examined the speed modulation of their firing rate (Fig. 7). Of the 287 EC2 principal neurons evaluated, 164 were physiologically classified as pyramidal cells, and 105 were classified as stellate cells (Fig. 7a–c). The proportions of p-Speed and n-Speed cells were similar between stellate cells and pyramidal cells (Fig. 7d,e). Consistent with a calcium imaging study that compared the speed representation of genetically labelled pyramidal and stellate cells^56^, and with an extracellular recording study that classified putative pyramidal cells and stellate cells using physiological criteria^77^, p-Speed putative pyramidal cells were found to have higher speed scores (Fig. 7f, P < 0.0001, *t*-test) and higher normalised speed slopes than p-Speed putative stellate cells (Fig. 7h, P < 0.05, *t*-test; also see Fig. 7g,i,j, Supplementary Tables S2 and S3). Furthermore, we found that p-Speed putative pyramidal cells, but not stellate cells, showed significant prospective speed representation (Fig. 7k and Supplementary Table S5, P < 0.05, *t*-test). p-Speed putative pyramidal cells represented speed more prospectively than p-Speed putative stellate cells (Fig. 7k; mean preferred temporal shifts of pyramidal cells, 103 ms; stellate cells, –87 ms, P < 0.05, *t*-test). Examining the relationship between the preferred theta phase and speed representation (Fig. 7l,m), we found that within pyramidal cells in EC2, p-Speed cells fired at an earlier theta phase than n-Speed cells (Fig. 7l). In summary, compared with p-Speed putative DG/CA3-projecting stellate cells, p-Speed putative CA1-projecting pyramidal cells represent speed more reliably and prospectively. Thus, the property of speed information transmitted from EC2 region to the target regions is pathway dependent.

### Speed representation in putative parvalbumin-expressing and somatostatin-expressing hippocampal interneurons

**Figure 8:**
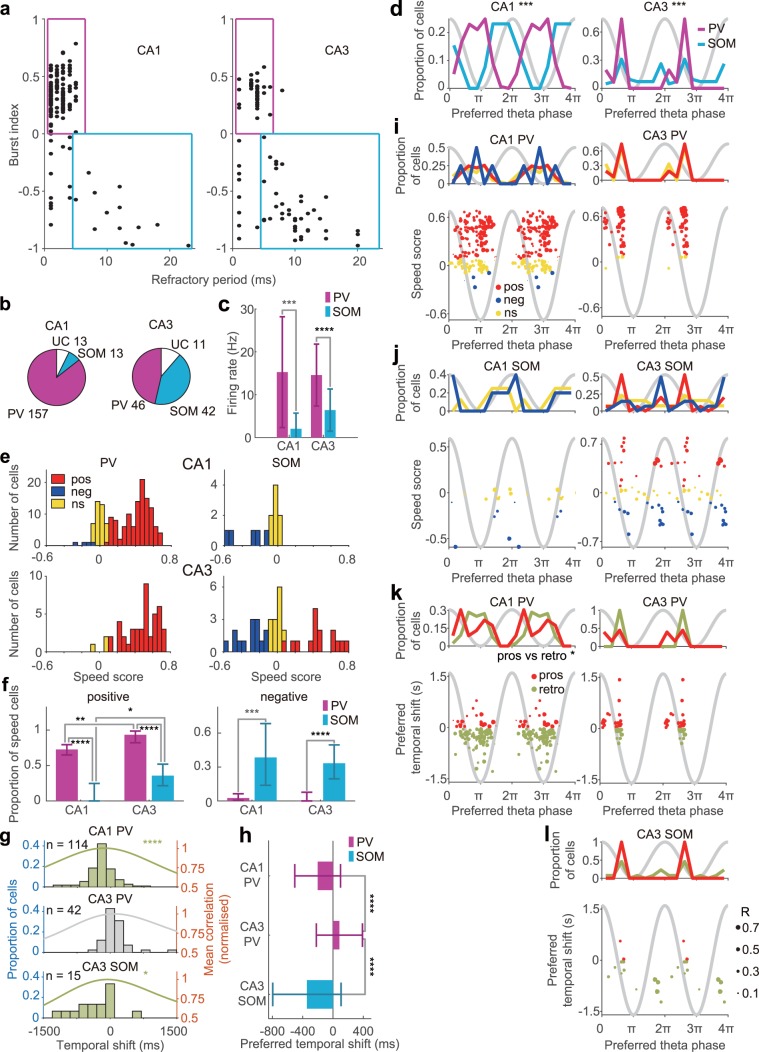
Speed representation of putative parvalbumin (PV)- and somatostatin (SOM)-expressing hippocampal interneurons. (**a**) Classification of putative PV-expressing and SOM-expressing interneurons in CA1 (left) and CA3 (right). The burst index and refractory period were used for the classification^47,80^. Each dot represents a single cell. Cells in magenta and light blue boxes were classified as PV and SOM interneurons, respectively. (**b**) The proportion of PV and SOM interneurons and unclassified (UC) interneurons in CA1 (left) and CA3 (right). Numbers indicate the number of each cell type. (**c**) Mean ( ± SD) firing rate of PV and SOM interneurons in CA1 (left) and CA3 (right). *t*-test, ***P < 0.001, ****P < 0.0001. (**d**) Distribution of the preferred theta phase of PV (magenta) and SOM (light blue) interneurons in CA1 (left) and CA3 (right). Watson U^2^ test, ***P < 0.001. (**e**) Distribution of speed scores of PV (left) and SOM (right) interneurons in CA1 (top) and CA3 (bottom). Pos, p-Speed cells. Neg, n-Speed cells. Ns, non-speed cells. (**f**) Proportion of p-Speed (positive, left) and n-Speed (negative, right) cells in PV and SOM interneurons in CA1 and CA3 ( ± 95% Clopper–Pearson confidence intervals). Fisher’s exact test with Bonferroni correction, *P < 0.05, **P < 0.01, ***P < 0.001, ****P < 0.0001. (**g**) Distribution of preferred temporal shifts (bars) and mean normalised correlations (lines) of p-Speed PV interneurons in CA1 (top), and p-Speed PV (middle) and SOM (bottom) interneurons in CA3. Light green, significantly retrospective representation, *t*-test, *P < 0.05, ****P < 0.0001. (**h**) Preferred temporal shifts of individual neurons (mean ± SD). ANOVA followed by Bonferroni test, ****P < 0.0001. (**i**) Distribution of the preferred theta phase (top) and the relationship between the speed score and preferred theta phase of p-Speed (pos, red), n-Speed (neg, blue) and non-speed (ns, yellow) CA1 (left) and CA3 (right) PV interneurons. (**j**) Same as (**i**) for SOM interneurons. (**k–l**) Distribution of the preferred theta phase (top) and the relationship between the preferred temporal shift and preferred theta phase (bottom) of individual prospective (pros, red) and retrospective (retro, light green) p-Speed CA1 (left) and CA3 (right) PV interneurons (**k**) and CA3 SOM interneurons (**l**). Note that there were no p-Speed CA1 SOM interneurons. Watson U^2^ test, *P < 0.05. (**i–l**) The size of each circle represents the strength of the theta locking (mean resultant length, *R*) of each neuron. Sinusoidal grey lines in (**d**) and (**i–l**); idealised theta oscillations recorded from EC3.

Many types of interneurons are known to exist in the hippocampus, and distinct types of interneurons are believed to have different roles in oscillatory network dynamics and information processing^78,79^. Soma-targeting parvalbumin (PV)-expressing interneurons and dendrite-targeting somatostatin (SOM)-expressing interneurons are the two main classes of hippocampal interneurons. A previous report optogenetically identified PV-expressing and SOM-expressing interneurons in the hippocampus and found that PV-expressing interneurons were ‘bursty’ with short refractory periods, while SOM-expressing interneurons were ‘non-bursty’ with longer refractory periods^80^. Therefore, we classified hippocampal interneurons into putative PV-expressing and SOM-expressing interneurons by using two physiological criteria, i.e., burst tendency and refractory period, parameters that have been shown to be reliable according to previous reports^47,80^ (Fig. 8a; see Methods section). Of 183 CA1 interneurons evaluated, 157 were classified as PV-expressing interneurons and 13 as SOM-expressing interneurons (Fig. 8b). Furthermore, 46 of 99 CA3 interneurons were classified as putative PV-expressing interneurons and 42 as SOM-expressing interneurons (Fig. 8b). Consistent with a previous report^80^, during exploration, putative PV-expressing interneurons had higher firing rates than putative SOM-expressing interneurons in both CA1 and CA3 (Fig. 8c). During waking theta oscillations, putative PV-expressing CA1 interneurons preferentially fired at the trough of theta, while putative SOM-expressing CA1 interneurons preferred the ascending phase of theta (Fig. 8d), as previously reported^80^. In CA3, putative PV-expressing interneurons fired preferentially at the descending theta phase while putative SOM-expressing interneurons, as a group, did not display a strong theta phase preference (Fig. 8d). In both CA1 and CA3, the proportions of p-Speed cells in putative PV-expressing interneurons were significantly higher than those in putative SOM-expressing interneurons (Fig. 8e,f,i,j; CA1, PV + cells, 114/157 = 72.6%, SOM + cells, 0/13 = 0.0%, P < 0.0001; CA3, PV + cells, 43/46 = 93.5%, SOM + cells, 15/42 = 35.7%, P < 0.0001, Fisher’s exact test with Bonferroni correction). In contrast, the proportions of n-Speed cells in putative SOM-expressing interneurons were significantly higher than those in putative PV-expressing interneurons (Fig. 8e,f,i,j; CA1, PV + cells, 4/157 = 2.5%, SOM + cells, 5/13 = 38.5%, P < 0.001; CA3, PV + cells, 0/46 = 0.0%, SOM + cells, 14/42 = 33.3%, P < 0.0001, Fisher’s exact test). The proportions of p-Speed cells in both putative PV-expressing and SOM-expressing cells were significantly higher in CA3 than in CA1 (Fig. 8f). Next, we compared the temporal shifts of putative PV-expressing interneurons and putative SOM-expressing interneurons. p-Speed putative PV-expressing CA1 interneurons and p-Speed putative SOM-expressing CA3 interneurons preferentially represented speed retrospectively (Fig. 8g,h,k,l), while p-Speed putative PV-expressing CA3 interneurons showed a trend of prospective speed representation (*t*-test, P = 0.079, Fig. 8g, Supplementary Table S5). Both p-Speed putative PV-expressing CA1 interneurons and p-Speed putative SOM-expressing CA3 interneurons represented speed significantly more retrospectively than p-Speed putative PV-expressing CA3 interneurons (Fig. 8h, ANOVA followed by Bonferroni test, P < 0.0001, Supplementary Table S5). In summary, putative PV-expressing and SOM-expressing hippocampal CA1 and CA3 interneurons represented speed differently.

## Discussion

In order to understand how speed information is handled in the hippocampal–entorhinal circuit, we compared the speed representation of distinct cell types across different sub-regions and layers of the hippocampus and MEC. Our results demonstrated that, in comparison with principal neurons, a larger proportion of putative interneurons are p-Speed cells in CA1, CA3, EC3, and EC5; which is consistent with the findings of other studies^10,48,55^. We also found that the speed score, speed slope, and speed information per second of the p-Speed interneurons were all higher than those of p-Speed principal neurons, suggesting the dominant role of interneurons in speed representation throughout the hippocampus-MEC circuit. It should be noted that, in both principal neurons and interneurons, the activity of neurons with higher-firing rates was more strongly correlated with speed (Fig. 2h), consistent with previous reports showing that the correlation coefficient between neuronal spike trains increases with firing rates^62,81^. Therefore, correlation between speed and firing rate may be an inherent property of the hippocampal-entorhinal circuit, and the apparent dominance of interneurons on speed representation may be, at least in part, the inevitable consequence of their higher firing rate.

The proportion of p-Speed cells in EC2 principal neurons was as large as that in EC2 interneurons. Furthermore, the proportion of p-Speed cells in the principal neurons of EC2 was larger than that in the hippocampus and EC3, suggesting the importance of EC2 for speed representation, as previously demonstrated^10^. However, the slopes of the regression lines calculated for speed and normalised instantaneous firing rates, as well as the speed information per spike (bits/spike) of p-Speed EC2 principal neurons, were lower than those of p-Speed CA1, CA3, and EC5 principal neurons. Finally, the speed scores, speed slopes, and speed information per second of p-Speed EC2 interneurons were all lower than, or similar to, those of p-Speed CA1, CA3, EC3, and EC5 interneurons. These results suggest that speed representation in the single p-Speed EC2 neurons is not more effective than those of other sub-regions/layers in the circuit.

Fully revealing prospective vs retrospective speed representation in each anatomical station of the hippocampal-entorhinal circuit is an important step towards understanding how speed information is processed in the circuit. It has been reported that the majority of speed cells in the MEC encode speed prospectively whereas those in the hippocampus encode speed retrospectively^10^. Our results are consistent with previous findings; we also revealed that most p-Speed EC2 interneurons represent speed prospectively, whereas p-Speed CA1 cells represent speed retrospectively. It has been suggested that the retrospective shift of hippocampal speed cells appears as an outlier^48^. However, our results revealed that most of the p-Speed EC3 and EC5 principal neurons also represent speed retrospectively. As a population, p-Speed CA3 cells, as well as p-Speed EC2 principal neurons and p-Speed EC3 and EC5 interneurons, did not show any significant bias towards either prospective or retrospective representation. Interestingly, the differences between the mean preferred temporal shifts of p-Speed EC2 principal neurons (7 ms) vs p-Speed EC2 interneurons (343 ms), p-Speed EC3 principal neurons (−254 ms) vs p-Speed EC3 interneurons (−50 ms), p-Speed EC2 principal neurons (7 ms) vs p-Speed EC3 principal neurons (−254 ms), and p-Speed EC3 interneurons (−50 ms) vs p-Speed EC2 interneurons (343 ms), were all significant and larger than expected in terms of axon conduction velocities and passive synaptic integration of the local circuit in superficial EC. Therefore, we hypothesise that the preferred temporal shifts are not simply inherited from ‘upstream neurons’ to ‘downstream neurons’ in the local circuit but rather reflect the interaction between local (within the MEC) and global (including outside of the MEC) circuit mechanisms. Furthermore, it was the p-Speed EC2 principal neurons and p-Speed EC3 interneurons, but not hippocampal and EC5 p-Speed cells or p-Speed EC2 interneurons and p-Speed EC3 principal neurons, that exhibited significant correlations between the magnitude of theta modulation and preferred temporal shift of speed representation. Therefore, the bias towards prospective and retrospective speed representation and their dependency on theta entrainment appear to be sub-region, layer, and cell-type specific.

As an animal moves through the place field, hippocampal place cells discharge at progressively earlier theta phases, a phenomenon known as phase precession^82^. As a result, when an animal traverses across place fields consisting of multiple place cells, the cell assembly representing the current location of the animal fires at the trough of the theta cycle, whereas those representing the previously and subsequently visited locations fire on the descending and ascending phases, respectively. Therefore, the neuronal sequence, in terms of a given behaviourally relevant time scale (in the order of seconds), is embedded in the ongoing theta rhythm (on the order of 100 ms) in a time-compressed manner, with retrospective and prospective spatial representation repeating in every theta cycle^62,83–85^. Moreover, running speed alters the sequence compression of place cell spikes^86^. Entorhinal grid cells also show phase precessions^58,87,88^, raising the possibility that the entorhinal cortex and hippocampus share the same theta compressed coding mechanism. In this study, we found that even in the principal neurons/interneurons in the same sub-region/layer, the preferred theta phases of neuronal spiking were correlated with the positive/negative speed modulation and preferred temporal shifts of speed representation in a sub-region/layer-specific manner. Further studies are required to determine how the sub-region/layer and cell type-specific theta-locked positive/negative and prospective/retrospective speed representation is linked to the sequence compression of spatial coding.

In EC2, calbindin-positive pyramidal cells are clustered and arranged in a hexagonal grid^76^ and project to CA1^75^, while calbindin-negative stellate cells are homogeneously distributed and project primarily to the DG^74,76^. The results of our study indicated that EC2 putative pyramidal cells, which project to the CA1 region, represented speed prospectively, while putative stellate cells, which project to the DG and CA3 regions, did not show significant temporal shifts of speed representation. These findings may be partly explained by a recent report demonstrating that the speed signal in VGluT2 neurons in the medial septum-diagonal band of Broca (MSDB) is integrated more effectively by pyramidal cells than stellate cells in the MEC^89^. Interestingly, p-Speed EC3 principal neurons, which project to CA1, represented speed retrospectively. Therefore, CA1 region receives prospective speed information from p-Speed EC2 pyramidal neurons and retrospective speed information from p-Speed EC3 principal neurons, as well as prospective speed information from p-Speed EC2 interneurons. How the prospective and retrospective speed information of p-Speed cells in the entorhinal cortex are integrated, and robust retrospective representation emerges in the CA1 region, remains elusive. Moreover, it is important to note that our conclusion rests on the validity and accuracy of the previously proposed classification procedure^77^, so it is important to confirm our results using genetically defined pyramidal and stellate cells^56,75,90^.

Because somatic and dendritic inhibitions are suggested to have distinct computational roles^78,79,91,92^, it is important to know how they are correlated with speed to fully understand how speed signals affect hippocampal computation. A recent calcium imaging study revealed that the activity of most PV-expressing and SOM-expressing CA1 interneurons increases with locomotion, relative to immobility, in a virtual reality track running task in head-fixed animals^60^. Interestingly, the activity of small and similar fractions of PV-expressing and SOM-expressing neurons decreases during locomotion and increases during periods of immobility^60^. In this study, we classified hippocampal interneurons as putative soma-targeting PV-expressing or dendrite-targeting SOM-expressing neurons by using physiological criteria based on burst tendency and refractory period^47^. In our analysis, we excluded the immobility periods ( < 2 cm/sec running speed) and examined the relationship between instantaneous firing rate vs running speed during locomotion. We found that most putative PV-expressing interneurons were p-Speed cells in both the CA1 (72.6%) and CA3 (93.5%) regions. However, none of the CA1 putative SOM-expressing interneurons and only 35.7% (15/42) of CA3 putative SOM-expressing interneurons were p-Speed cells. In contrast, only a negligible fraction of putative PV-expressing interneurons in the hippocampus exhibited negative speed modulation, whereas approximately one-third of SOM-expressing hippocampal interneurons exhibited negative speed modulation. CA1 place cells have been shown to fire at the peak of theta when the animal is at the entrance of a place field, fire maximally at the trough of theta when the animal is in the centre of the field, and fire near the peak of theta when the animal is at the exit of the place field^47,58,62,63^. Our results suggest that CA1 place cells receive positive speed-modulated inhibition perisomatically during the trough of theta (i.e. the theta phase at which the cells fire in the centre of the place field) (Fig. 8i, left), and negative speed-modulated inhibition at the dendrites near the peak of theta (i.e. the theta phase at which the cells fire at the entrance and exit of the place field) (Fig. 8j, left). A similar argument may be true for the CA3 region. Indeed, it has been demonstrated that CA3 place cells fire at the ascending phase/trough of theta when the animal is at the entrance of the place field, fire maximally during the descending phase of theta when the animal is at the centre of the field, and fire near the peak of theta oscillations when the animal is at the exit of the place field^47^. Our results suggest that CA3 place cells receive positive speed-modulated inhibition perisomatically during the descending phase of theta (i.e. the theta phase at which the cells fire in the centre of the place field) (Fig. 8i, right), whereas these cells receive negative speed-modulated inhibition at the dendrites during the peak of theta (i.e. the theta phase at which the cells fire at the exit of the place field) (Fig. 8j, right). These observations can constrain computational modelling to explain how spatial representation and speed signals are linked through theta oscillations in the hippocampus.

Furthermore, we found that p-Speed CA1 putative PV-expressing interneurons and p-Speed CA3 SOM-expressing interneurons represented speed retrospectively, while p-Speed CA3 PV-expressing interneurons showed a trend for prospective speed representation. Collectively, our results suggest that CA1 pyramidal neurons receive positive speed-modulated retrospective inhibition perisomatically at the trough of theta, whereas CA3 pyramidal neurons receive positive speed-modulated prospective inhibition perisomatically and positive speed-modulated retrospective inhibition at the dendrites. However, the physiological importance of perisomatic vs dendritic speed-modulated inhibition warrants further investigation. We did not attempt to classify interneurons in the entorhinal cortex into putative PV-expressing and SOM-expressing cells because, to our knowledge, there are no established criteria for distinguishing PV-expressing and SOM-expressing interneurons in the MEC using extracellular physiological data. Since PV-expressing and SOM-expressing interneurons distinctly control spatial coding in the MEC^93^, it would be interesting to characterise speed representation in genetically defined PV-expressing and SOM-expressing interneurons in the MEC^67^ in the future.

On balance, our results indicate that distinct cell types at each anatomical station in the hippocampal–entorhinal circuit represent speed information differently. Neuronal activity is positively modulated by locomotion speed in the visual cortex^94^, ventral tegmental area^95–97^, medial mammillary nucleus^98^, interpeduncular nucleus and habenula^99^, subiculum and post-subiculum^100^, and medial septum^101,102^. Above all, medial septal neurons appear to play a significant role in controlling speed representation in the hippocampus-entorhinal cortex (also see Dannenberg *et al*.^19^ and Carpenter *et al*.^103^). Most medial septal neurons display theta-locked firing patterns during locomotion^101,102^. VGluT2 neurons in MSDB are the main effector cells of locomotion, and the concerted action of VGluT2 neurons and other MSDB cell types are essential for theta oscillations^104^. Pharmacological inactivation of MSDB cells impairs the ability to estimate linear distances based on self-motion information^105^ and self-motion-generated place fields but not landmark-tied place fields in the hippocampus^106^. Systemic administration of the muscarinic antagonist scopolamine flattens the positive correlation between running speed and entorhinal theta frequency in rats^107^ and reduces spatial tuning of grid cells but not the tuning of head-direction cells in the MEC^108^. The removal of MS input resulted in strengthening of the firing-rate speed signals, while decreasing the strength of the oscillatory speed signals, suggesting that firing rate and oscillatory speed signals, which are distinctly modulated by MS input, co-exist in the entorhinal cortex^54^. How speed representation in each cell type in each sub-region/layer of the hippocampus-entorhinal cortex emerges from local and global circuit mechanisms, and if/how the speed information is utilised to update the representation of the current location of the animal and to guide behaviour, need further investigation.

## Methods

Data from four male Long-Evans rats (rat ID; ec012, ec013, ec014 and ec016; 250–400 g), which were obtained from previous studies^58,61–64^, were analysed. Speed cells were not reported in the previous studies. The experiments were approved by the Institutional Animal Care and Use Committee of Rutgers University. All procedures for animal care and use were performed in accordance with the National Institutes of Health *Guide for the Care and Use of Laboratory Animals*. The data were deposited at CRCNS (Collaborative Research in Computational Neuroscience - Data sharing; https://crcns.org/data-sets/hc/hc-3/about-hc-3) and the Buzsáki lab website (https://buzsakilab.nyumc.org/datasets/MizusekiK/). A detailed description of the experimental procedures, the histological localisation of recording sites, behavioural testing, data collection and spike sorting can be found in previous publications^57,58^. Below, we describe the analytical methods used in this study and relevant experimental procedures (animals and surgery, localisation of recording sites, data collection and analysis, and classification of principal neurons and interneurons) from the original publication^58^.

### Animals and surgery

Four male Long-Evans rats (250–400 g) were subjected to isoflurane anaesthesia (1–1.5%) and implanted with a 4-shank silicon probe in the right dorsocaudal MEC. In three of the rats, an additional silicon probe was implanted in the right dorsal hippocampus. The individual silicon probes were attached to respective micromanipulators and moved independently. The silicon probe in the entorhinal cortex was moved gradually to the desired locations over the course of 4–12 weeks while recordings were also made in the pyramidal layer of CA1 or CA3. The hippocampal probe consisted of 4 or 8 shanks (with a 200-µm separation between shanks). Each shank had 8 recording sites (160 µm^2^ for each site; 1–3 MΩ impedance), which were staggered to provide a two-dimensional arrangement (with a vertical separation of 20 µm). The shanks were aligned parallel to the septo-temporal axis of the hippocampus (45 ° parasagittal), positioned centrally at 3.5 mm posterior from bregma and 2.5 mm lateral from the midline. The probe in the entorhinal cortex had 4 shanks and was positioned such that the different shanks recorded from different layers (4.5 mm lateral from the midline; 0.1 mm anterior to the edge of the transverse sinus at an angle of 20 °–25 ° in the sagittal plane with the tip pointing in the anterior direction). Two stainless steel screws inserted above the cerebellum were used as indifferent and ground electrodes during recordings.

### Localisation of recording sites

To facilitate track identification, DiI was applied to the back of the shanks before implantation. To identify the location of the recording sites, a small anodal DC current (2 µA for 10 sec) was passed through the platinum-iridium recording pad of the probe 1 or 2 days before sacrificing the animals. The rats were deeply anaesthetised and perfused through the heart first with 0.9% saline solution followed by 10% formalin solution. The brains were sectioned using a Vibratome (Leica, Germany) at 100 µm in the coronal plane for the hippocampus, and in the sagittal plane for the MEC. The sections were mounted on slides, Nissl-stained, and mounted on coverslips. The tracks of the silicon probe shanks were reconstructed from multiple sections. The combination of these labelling and histological methods, magnitude of silicon probe movement between sessions by the experimenter (~20% of shrinkage as a result of the histological procedure was taken into account), and local field potential (LFP) patterns characteristic to certain layers^109–111^ allowed for the post-mortem identification of the tracks and recording sites^58^. Histological analysis, including histological photographs, has been reported previously in detail^58^.

The locations of the silicon probe shanks were identified by the reversal of ripple-triggered sharp waves^110^ and reversal of theta waves^87,111–113^ assisted by the histological verification of the recording tracks, as previously reported in detail^58^. Each MEC neuron was assigned to a cortical layer based on the location at which the spike waveform was recorded on the silicon probes^58^.

### Data collection and analysis

After recovery from surgery (~1 week postoperatively), physiological signals were recorded during open-field tasks in which the rats chased randomly dispersed drops of water or pieces of Froot Loops (~25 mg, Kellogg’s) on an elevated square platform (180 cm × 180 cm or 120 cm × 120 cm). During the recording sessions, neurophysiological signals were amplified (1,000 × ), bandpass-filtered (1–5,000 Hz) and acquired continuously at 20 kHz on a 128-channel DataMax system (16-bit resolution; RC Electronics). After recording, the LFP was down-sampled to 1250 Hz for additional analysis. Positive polarity was up throughout this study. For offline spike sorting, wideband signals were digitally high-pass filtered (0.8 kHz–5 kHz) and the waveforms were re-sampled^114^. Spike sorting was performed automatically, using ‘KlustaKwik’^115^ (http://klustawik.sourceforge.net), followed by manual adjustment of the clusters (using ‘Klusters’^116^ software package; http://klusters.sourceforge.net). Only units with clear refractory periods and well-defined cluster boundaries were included in our analyses^115^. The tip of the probe either moved spontaneously between sessions or was moved by the experimenter. However, we cannot exclude the possibility that some neurons recorded in different sessions were identical because spikes from sessions recorded on different days were clustered separately. Therefore, the total number of independent neurons may be less than the numbers shown in Supplementary Table S1.

Theta periods were detected automatically using the ratio of the power in the theta band (5–11 Hz) to the power of nearby bands (1–4 Hz, 12–14 Hz) of the EC3 LFP, followed by manual adjustment with the aid of visual inspection of whitened power spectra (using a low-order autoregressive model^117^) and the raw traces^118^. To determine the phase of filtered theta waves, only theta epochs during walking were used for analysis. The instantaneous theta phase was derived from the Hilbert transform of the band-pass filtered LFP trace. The phase was then unwrapped into a mostly monotonically increasing signal by adding 2π at every phase reset. The phase at which the spike occurred was obtained from the unwrapped theta phase using linear interpolation followed by a modulo 2π operation. For theta phase analyses, peaks = 0, 2π and troughs = π throughout this study. The mean direction and mean resultant length of the theta phases of a given neuron’s spikes were taken as the preferred phase and the strength of theta locking of that neuron.

To track the position of the animal, images from two small light-emitting diodes (LEDs, aligned in the midline in the front-back direction with a separation of 10–15 cm) mounted above the head-stage were recorded by a digital video camera placed directly above the experimental apparatus and recorded with a sampling frequency of 30 Hz. The sampling resolution was such that a pixel was approximately equivalent to 0.4 cm. For each video frame, the area at the front LED was detected using the light intensity threshold, and the centre of mass of the front area of the LED (typically > 20 pixels) was regarded as the position of the animal. The position was linearly interpolated in 25.6 ms bins and then smoothed by Matlab’s ‘smooth.m’ function using a width of 281.6 ms (11 bins) and local regression with weighted linear least squares and a first-degree polynomial model^48^.

### Classification of principal neurons and interneurons

Hippocampal principal neurons and interneurons were separated on the basis of their auto-correlograms, waveforms, and mean firing rates^85,114^. As there are no generally accepted methods for the segregation of MEC principal neurons and interneurons^10,48,87,119–124^, we sought an alternative approach by taking advantage of the simultaneously recorded cells in the MEC to physiologically identify the recorded units as excitatory or inhibitory neurons using their short-latency temporal interactions with other neurons^118,125,126^. For identifying excitatory and inhibitory connections between neurons, short-latency, short-duration sharp peaks/troughs in the cross-correlograms were used^55,119,124–129^. Monosynaptic connections between pairs of units were detected using a non-parametric significance test based on jittering of spike trains as described previously in detail^126^. Briefly, for each cell pair, each spike of each neuron in the original data set was randomly and independently perturbed (or ‘jittered’) on a uniform interval of [−5, + 5] ms, to form a surrogate data set. The process was repeated independently 1,000 times to form 1,000 such surrogate data sets. The cross-correlograms were then constructed for surrogate data sets as a function of latency across the interval [−20, + 20] msec. Global bands at an acceptance level of 99% were constructed for the cross-correlogram from the maximum and minimum of each jitter surrogate cross-correlogram across the interval [−20, + 20] msec. The short-latency peak in the original cross-correlogram was determined to be statistically significant (at P < 0.01) when the counts in the cross-correlogram were atypical with respect to the upper global band anywhere at the latency [1,5] msec^126^. Similarly, short-latency significant troughs were considered as a result of inhibition when at least one 1-msec bin was significantly depressed (P < 0.01) anywhere at the latency [1,5] msec. For cell pairs recorded from the same electrode, the 0–1 ms bin was not considered, because our clustering programme cannot resolve superimposed spikes.

The excitatory and inhibitory neurons physiologically identified by the jittering method, in turn served as a template for exploring other spike features. For each unit, various parameters were calculated, including the trough to peak latency of the filtered (0.8 kHz–5 kHz) spike waveform, half-amplitude width, asymmetry index (ratio of the difference between right and left baseline-to-peak amplitudes and their sum), firing rate, and features of the auto-correlogram. Next, we explored the multi-dimensional space formed by these parameters for the subset of units identified as inhibitory or excitatory based on cross-correlogram analysis as described above. As in the neocortex^118^, the combination of trough to peak latency and the asymmetry index of the filtered (0.8 kHz–5 kHz) spike waveform provided the best separation between the excitatory and inhibitory neurons physiologically identified by the jittering method^58^. We used the hyperplane that divided the physiologically identified excitatory and inhibitory neurons to separate units into putative principal neurons and putative interneurons^58^.

### Classification of putative pyramidal cells and stellate cells in the MEC layer 2

We used the previously published classification procedure^77^ to classify putative principal neurons in the MEC layer 2 into putative pyramidal cells and stellate cells by using extracellular physiological data. The strength of theta phase locking and preferred theta phase with respect to the local theta oscillations recorded in the MEC layer 2 were used for the classification. We used the code and parameters that were deduced from calbindin-labelling experiments and published previously^77^.

### Classification of putative PV and SOM-expressing hippocampal interneurons

Following guidelines from previous publications^47,80^, hippocampal interneurons were classified into two groups, i.e., putative PV-expressing and SOM-expressing interneurons, according to the burst index and refractory period. A burst index and a refractory period were calculated from the spike auto-correlogram (1 ms time bin) of each cell. To calculate a burst index, first we estimated the amplitude of the burst from the spike auto-correlogram by subtracting the mean value between 40 and 50 ms (baseline) from the peak measured between 0 and 10 ms. A burst index was defined as the amplitude of the burst divided by the larger of the peak and the baseline so that the indexes ranged from −1 to 1^80^. To calculate the refractory period, first the instantaneous derivative from 0 ms to the time of the peak of the auto-correlogram was calculated and the SD of the derivative values was estimated. The refractory period was defined as the first bin for which the instantaneous derivative exceeded one SD of the derivative values^47,80^. Interneurons with a positive burst index and a refractory period shorter than 7 ms were classified as putative PV-expressing interneurons, while those with a negative burst index and a refractory period longer than 4 ms were classified as putative SOM-expressing interneurons^47,80^.

### Theta index and theta-modulated cells

Following guidelines from a previous publication^10^, we classified cells into theta-modulated cells and non-modulated cells by the theta index^130^. The theta index was calculated from the spike auto-correlogram (5-ms time bin) of each cell, using spikes that occurred during theta epochs with walking/running ( > 2 cm/sec running speed). The theta index was defined as the peak (the mean of auto-correlogram between 100 and 140 ms) minus the trough (the mean of auto-correlogram between 50 and 70 ms) divided by their sum^130^. Cells with a theta index larger (or smaller) than 0.2 were classified as theta-modulated (or non-modulated) cells^10^.

### Analysis of speed cells

The methods used to analyse speed cells were adapted from a previous study^10^. Neuronal spikes were sorted into 25.6-ms bins. The instantaneous firing rate was obtained by dividing the numbers of spikes for each neuron by the bin size and smoothed with a Gaussian filter (SD, 512 ms). The instantaneous running speed was calculated by dividing the distance of the animal’s smoothed position (see ‘Data collection and analysis’ above**)** between adjacent bins by bin size (25.6 ms) and smoothed with a Gaussian filter (SD, 512 ms). We compared the effect of smoothing filters using different sizes of Gaussian filters (SDs, 128, 256, 512, and 1,024 ms) for both smoothing instantaneous firing rate and running speed (Fig. 1d–f) and obtained very similar results. Other than Fig. 1d–f, we showed the results of using a Gaussian filter with an SD of 512 ms. Periods while the rat ran slower than 2 cm/s and faster than 50 cm/s were removed from the analysis. A speed score was defined for each cell as the Pearson product-moment correlation coefficient between the cell’s instantaneous firing rate and the rat’s instantaneous running speed, on a scale from −1 to 1^10^. Chance-level statistics were constructed by a shuffling procedure where the spike train was circularly time-shifted relative to the rat’s position by a random interval between 30 s and the length of the session minus 30 s, with the end of the trial wrapped to the beginning. Shuffling was repeated 100 times for each cell. A cell was defined as a positive speed cell if its speed score exceeded the 99th percentile of distribution of speed scores from the shuffled data from all cells in the hippocampus and MEC and as a negative speed cell if its speed score was lower than the 1st percentile of distribution of speed scores from the shuffled data from all cells in the hippocampus and MEC.

A speed slope was defined as the slope of the least-squares linear regression between the instantaneous firing rate of each cell and instantaneous running speed of the rat. A normalised speed slope was defined as the slope of the least-squares linear regression between the instantaneous firing rate divided by the mean firing rate for each cell and the instantaneous running speed of the rat.

### The temporal bias of p-speed cells

We calculated the Pearson product-moment correlation coefficients between the running speed and instantaneous firing rate that was time-shifted in a step of 25.6 ms at the interval between −1,536 ms and 1,536 ms. The temporal shift that maximised the correlation coefficient was termed ‘preferred temporal shift’ of that neuron. The p-Speed cells whose preferred temporal shifts were either −1,536 ms or 1,536 ms were removed for further analyses. To calculate the mean normalised correlation as a function of temporal shift, for each neuron, correlation coefficients as a function of temporal shift divided by the correlation coefficient at the zero temporal shift (the ‘speed score’ of that neuron) were defined as a normalised correlation; the mean normalised correlation was calculated for each cell type in each sub-region/layer.

### Speed information

According to the spatial information content introduced in a previous publication^72^, we defined speed information content as follows. First, for each neuron, the tuning curve of the firing rate vs speed was constructed using bins of 4 cm/s, from 2 cm/s to 50 cm/s. Speed information was defined as follows:

$${\rm{Speed}}\,\text{information}\,\text{per}\,\text{spike}=\mathop{\sum }\limits_{i=1}^{N}{p}_{i}\frac{{\lambda }_{i}}{\lambda }{\log }_{2}(\frac{{\lambda }_{i}}{\lambda })$$

$$\text{Speed}\,\text{information}\,\text{per}\,\text{second}\,=\,\mathop{\sum }\limits_{i=1}^{N}{p}_{i}{\lambda }_{i}{\log }_{2}(\frac{{\lambda }_{i}}{\lambda })$$

where *i* represents speed bin identification number, *p*_*i*_ is the probability of occupancy of the *i*-th bin, *λ*_*i*_ is the mean firing rate of the *i*-th bin, and *λ* is the overall mean firing rate of the cell with speed between 2 cm/s and 50 cm/s.

### Spatial information, head directional information, and gridness score

To compute spatial information, head directional information, and gridness score, we used periods with a running speed of > 2 cm/s to filter out the static periods. To construct a firing-rate map in the open field, the position and spiking data were sorted into bins of 3 × 3 cm, generating raw maps of occupancy and spike number. The occupancy and spike number maps were individually smoothed by applying a Gaussian kernel (SD, 3 cm). Smoothed firing rate map was constructed by dividing the smoothed spike number map by the smoothed occupancy map. Spatial information^72^ was defined as follows:

$$\text{Spatial}\,\text{information}\,\text{per}\,\text{spike}\,=\,\mathop{\sum }\limits_{i=1}^{N}{p}_{i}\frac{{\lambda }_{i}}{\lambda }{\log }_{2}(\frac{{\lambda }_{i}}{\lambda })$$

$$\text{Spatial}\,\text{information}\,\text{per}\,\text{second}\,=\,\mathop{\sum }\limits_{i=1}^{N}{p}_{i}{\lambda }_{i}{\log }_{2}(\frac{{\lambda }_{i}}{\lambda })$$

where *i* represents spatial bin identification number, *p*_*i*_ is the probability of occupancy of the *i*-th bin, *λ*_*i*_ is the mean firing rate of the *i*-th bin, and *λ* is the overall mean firing rate of the cell on the open field.

The degree of spatial periodicity was quantified following previous reports^10,131,132^. Briefly, the autocorrelogram of firing rate map was calculated based on Pearson’s product moment correlation coefficient with corrections for edge effects and unvisited locations. With *λ (x, y*) denoting the average firing rate of a cell at location *(x, y)*, the autocorrelation between the fields with spatial lags of *τ*_*x*_ and *τ*_*y*_ was estimated as:

$$r({\tau }_{x},\,{\tau }_{y})=\frac{n{\sum }^{}\lambda (x,y)\lambda (x-{\tau }_{x},\,y-{\tau }_{y})-{\sum }^{}\lambda (x,y){\sum }^{}\lambda (x-{\tau }_{x},\,y-{\tau }_{y})}{\sqrt{n{\sum }^{}\lambda {(x,y)}^{2}-{({\sum }^{}\lambda (x,y))}^{2}\,}\sqrt{n{\sum }^{}\lambda {(x-{\tau }_{x},y-{\tau }_{y})}^{2}-{({\sum }^{}\lambda (x-{\tau }_{x},y-{\tau }_{y}))}^{2}}}$$

where the summation is over all *n* bins in *λ (x, y)* for which firing rate was estimated for both *λ (x, y)* and *λ (x* – *τ*_*x*_*, y* – *τ*_*y*_*)*. Autocorrelations were not estimated for lags of *τ*_*x*_, *τ*_*y*_ where *n* < 20. A ‘gridness score’ for each cell was determined from a series of expanding circular samples of the autocorrelogram, each centred on the central peak but with the central peak excluded^10,131,132^. The radius of the central peak was defined as either the first local minimum in a curve showing correlation as a function of average distance from the centre, or as the first incidence where the correlation was under 0.2, whichever occurred first^10,132^. The radius of the successive circular samples was increased in steps of 1 bin (2.5 cm) from a minimum of 10 cm more than the radius of the central peak, to a maximum of 10 cm less than the width of the box. For each sample, we calculated the Pearson correlation coefficient of the ring with its rotation in α degrees, first for angles of 60 ° and 120 °, and then for angles of 30 °, 90 °, and 150 °. The lowest correlation coefficient in the first group (60 ° and 120 °) minus the largest correlation coefficient in the second group (30 °, 90 °, and 150 °) was defined as the minimum correlation difference of the sample. The gridness score was defined as the highest minimum correlation difference in the entire set of successive circular samples^10,131,132^.

The head direction was defined based on the positions of front and rear LEDs. Head direction and spiking data were sorted in bins with 6 ° size. Directional firing rate map was constructed by dividing the spike number by the occupancy for each bin. According to the spatial information content introduced in a previous publication^72^, we defined the directional information as follows:

$$\text{Directional}\,\text{information}\,\text{per}\,\text{spike}\,=\,\mathop{\sum }\limits_{i=1}^{N}{p}_{i}\frac{{\lambda }_{i}}{\lambda }{\log }_{2}(\frac{{\lambda }_{i}}{\lambda })$$

$$\text{Directional}\,\text{information}\,\text{per}\,\text{second}\,=\,\mathop{\sum }\limits_{i=1}^{N}{p}_{i}{\lambda }_{i}{\log }_{2}(\frac{{\lambda }_{i}}{\lambda })$$

where *i* represents directional bin identification number, *p*_*i*_ is the probability of occupancy of the *i*-th bin, *λ*_*i*_ is the mean firing rate of the *i*-th bin, and *λ* is the overall mean firing rate of the cell on the open field.

The data were analysed using custom-written MATLAB-based software.

## Supplementary information

Supplementary Information.

## Data Availability

The data are available at CRCNS (https://crcns.org/data-sets/hc/hc-3/about-hc-3) and the Buzsáki lab website (https://buzsakilab.nyumc.org/datasets/MizusekiK/).
