# Supplementary material for: Cell type, sub-region, and layer-specific speed representation in the hippocampal–entorhinal circuit
Source: Sci Rep. 2020 Jan 29;10:1407. doi: 10.1038/s41598-020-58194-1 (PMC6989659; doi:10.1038/s41598-020-58194-1)
Supplement: Supplementary file 1 — Supplementary Information. [file 41598_2020_58194_MOESM1_ESM.pdf]

Supplementary information

Cell type, sub-region, and layer-specific speed representation in the hippocampal–entorhinal circuit

Motosada Iwase, Takuma Kitanishi, and Kenji Mizuseki

Abbreviations

MEC: medial entorhinal cortex

EC2: medial entorhinal cortex layer 2

EC3: medial entorhinal cortex layer 3

EC5: medial entorhinal cortex layer 5

PV: parvalbumin

SOM: somatostatin

p-Speed cells: positive speed cells

n-Speed cells: negative speed cells

Table S1. Number of cells analysed, and numbers and proportions of p-Speed, n-Speed, and non-speed cells.

(a) **Number of recorded cells**

	Principal neurons	Interneurons	Unclassified cells	All cells
CA1	1266	183	96	1545
CA3	378	99	20	497
EC2	287	51	14	352
EC3	311	113	44	468
EC5	357	51	85	493

(b) **Number of speed cells (Proportion of speed cells)**

Principal neurons

	p-Speed cells	n-Speed cells	Non-speed cells	Principal neurons
CA1	299 (23.6%)	13 (1.0%)	954 (75.4%)	1266
CA3	62 (16.4%)	33 (8.7%)	283 (74.9%)	378
EC2	92 (32.1%)	10 (3.5%)	185 (64.5%)	287
EC3	56 (18.0%)	29 (9.3%)	226 (72.7%)	311
EC5	109 (30.5%)	38 (10.6%)	210 (58.8%)	357

Interneurons

	p-Speed cells	n-Speed cells	Non-speed cells	Interneurons
CA1	118 (64.5%)	14 (7.7%)	51 (27.9%)	183
CA3	63 (63.6%)	18 (18.2%)	18 (18.2%)	99
EC2	22 (43.1%)	7 (13.7%)	22 (43.1%)	51
EC3	69 (61.1%)	4 (3.5%)	40 (35.4%)	113
EC5	32 (62.7%)	5 (9.8%)	14 (27.5%)	51

Principal neurons and interneurons

	p-Speed cells	n-Speed cells	Non-speed cells	Principal neurons + Interneurons
CA1	417 (28.8%)	27 (1.9%)	1005 (69.4%)	1449
CA3	125 (26.2%)	51 (10.7%)	301 (63.1%)	477
EC2	114 (33.7%)	17 (5.0%)	207 (61.2%)	338
EC3	125 (29.5%)	33 (7.8%)	266 (62.7%)	424
EC5	141 (34.6%)	43 (10.5%)	224 (54.9%)	408

All neurons

	p-Speed cells	n-Speed cells	Non-speed cells	All cells
CA1	435 (28.2%)	35 (2.3%)	1075 (69.6%)	1545
CA3	133 (26.8%)	51 (10.3%)	313 (63.0%)	497
EC2	115 (32.7%)	18 (5.1%)	219 (62.2%)	352
EC3	142 (30.3%)	36 (7.7%)	290 (62.0%)	468
EC5	165 (33.5%)	46 (9.3%)	282 (57.2%)	493

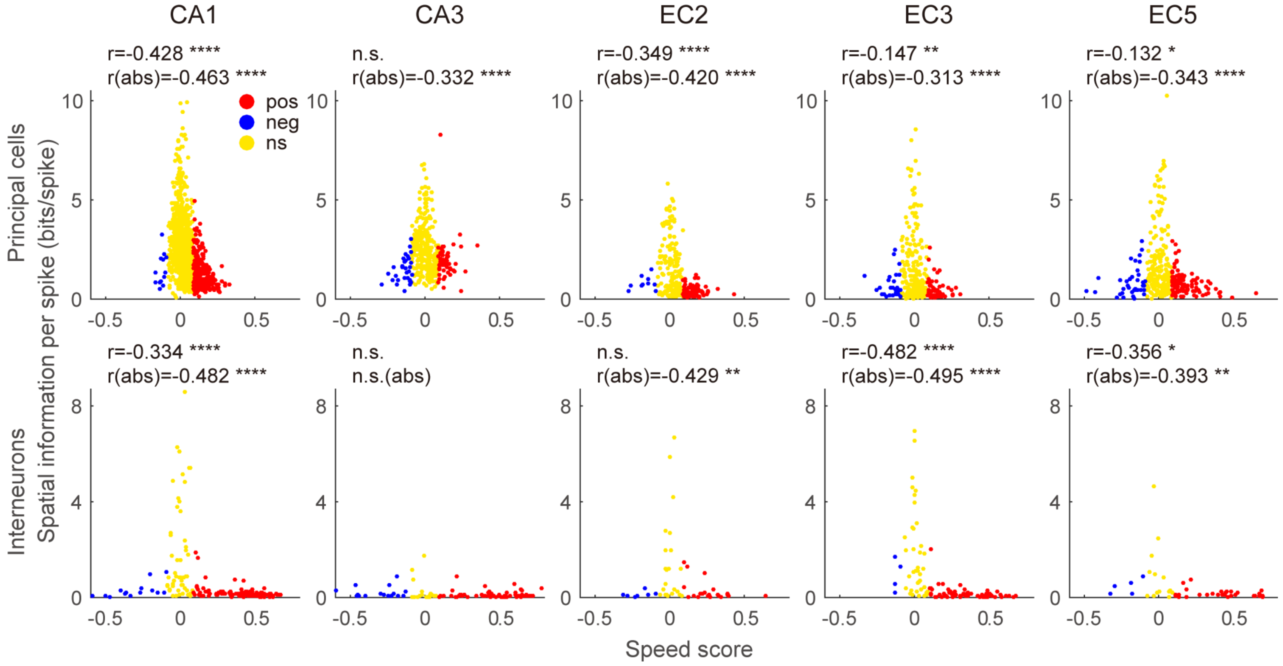

Figure S1. Relationship between spatial information per spike (bits/spike) and speed score.

Spatial information per spike (bits/spike) and speed score of individual neurons are shown. Principal neurons (top) and interneurons (bottom) of CA1, CA3, EC2, EC3, and EC5 are shown separately. r, correlation coefficient between spatial information per spike (bits/spike) and speed score. r(abs), correlation coefficient between spatial information per spike (bits/spike) and absolute value of speed score. * $P < 0.05$, ** $P < 0.01$, **** $P < 0.0001$. n.s., the correlation between spatial information per spike (bits/spike) and speed score is not statistically significant. n.s. (abs), the correlation between spatial information per spike (bits/spike) and absolute value of speed score is not significant. Red, p-Speed cells (pos); Blue, n-Speed cells (neg); Yellow, non-speed cells (ns). Consistent with the previous study[1], overall we observed that spatial information per spike (bits/spike) and (absolute value of) speed score were negatively correlated.

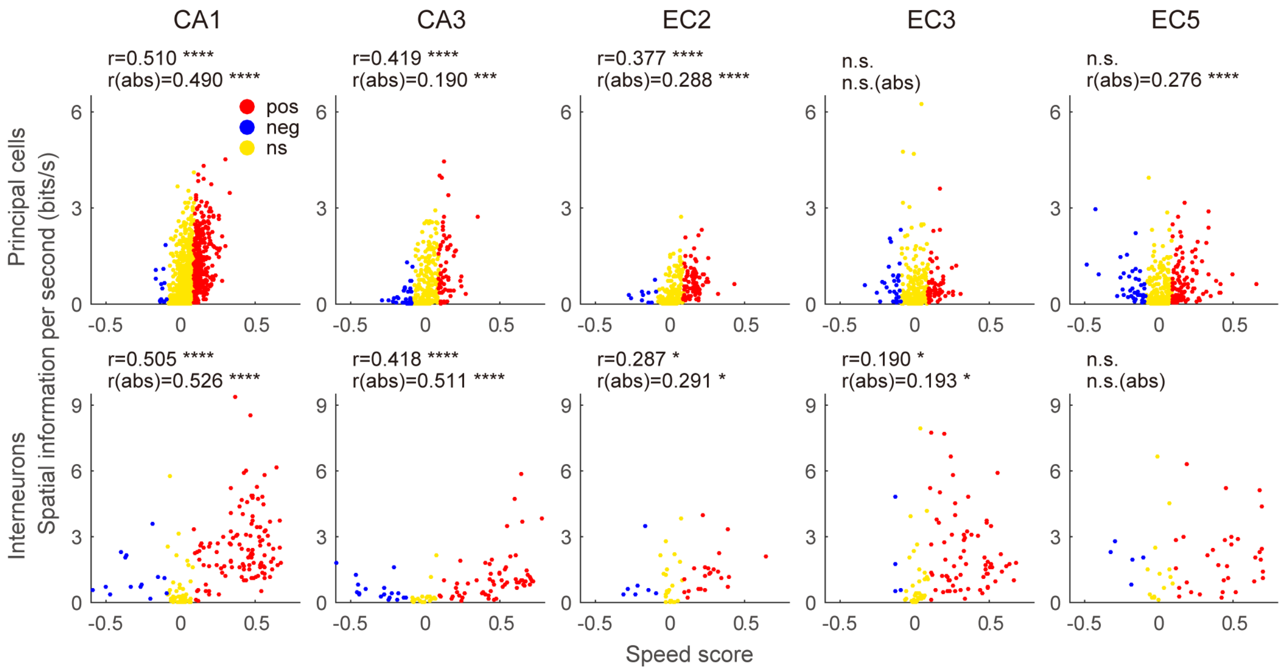

Figure S2. Relationship between spatial information per second (bits/s) and speed score.

Spatial information per second (bits/s) and speed score of individual neurons are shown. Principal neurons (top) and interneurons (bottom) of CA1, CA3, EC2, EC3, and EC5 are shown separately. r, correlation coefficient between spatial information per second (bits/s) and speed score. r(abs), correlation coefficient between spatial information per second (bits/s) and absolute value of speed score. * P < 0.05, *** P < 0.001, **** P < 0.0001. n.s., the correlation between spatial information per second (bits/s) and speed score is not significant. n.s. (abs), the correlation between spatial information per second (bits/s) and absolute value of speed score is not significant. Red, p-Speed cells (pos); Blue, n-Speed cells (neg); Yellow, non-speed cells (ns). Overall, we found that spatial information per second (bits/s) and (absolute value of) speed score positively correlated. The discrepancy between the relationship between spatial information per spike (bits/spike) vs speed score (Fig. S1) and the relationship between spatial information per second (bits/s) vs speed score (this figure) can be partly explained by the positive correlation between firing rate and (absolute value of) speed score (Fig. 2h and in the Results).

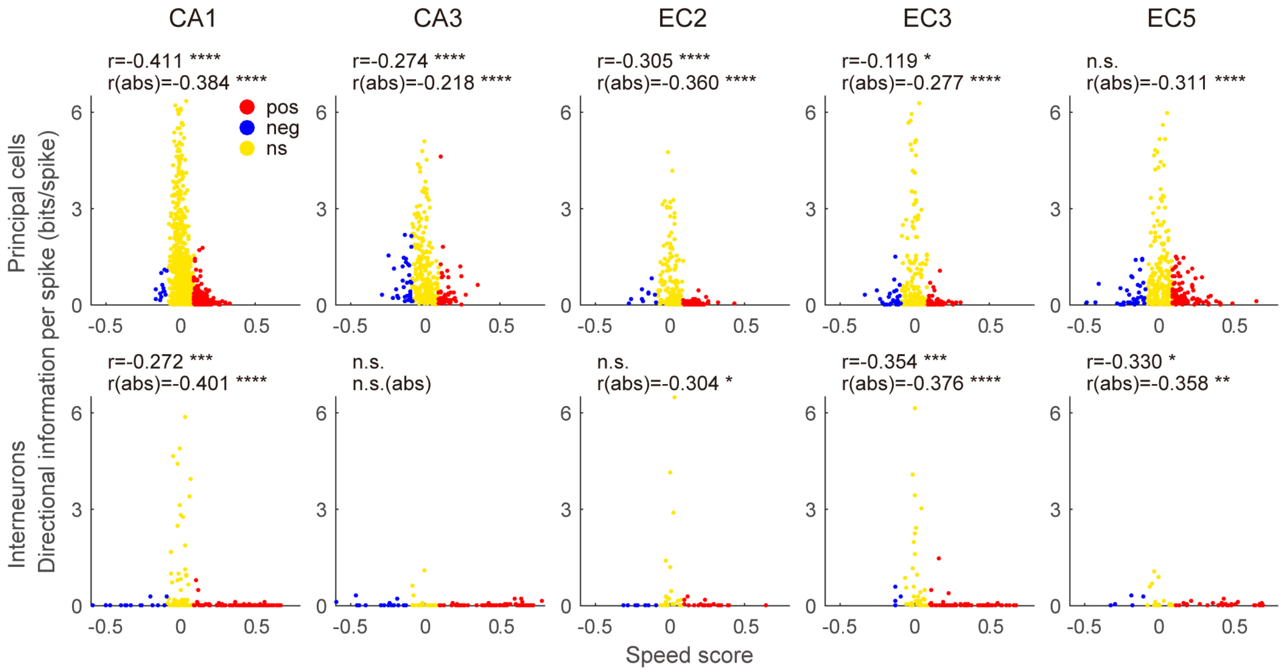

Figure S3. Relationship between directional information per spike (bits/spike) and speed score.

Directional information per spike (bits/spike) and speed score of individual neurons are shown. Principal neurons (top) and interneurons (bottom) of CA1, CA3, EC2, EC3, and EC5 are shown separately. r, correlation coefficient between directional information per spike (bits/spike) and speed score. r(abs), correlation coefficient between directional information per spike (bits/spike) and absolute value of speed score. * $P < 0.05$, ** $P < 0.01$, *** $P < 0.001$, **** $P < 0.0001$. n.s., the correlation between directional information (bits/spike) and speed score is not significant. n.s. (abs), the correlation between directional information per spike (bits/spike) and absolute value of speed score is not significant. Red, p-Speed cells (pos); Blue, n-Speed cells (neg); Yellow, non-speed cells (ns). Consistent with the previous study reporting the relationship between head direction score and speed score[1], overall we found that directional information per spike (bits/spike) and (absolute value of) speed score negatively correlated.

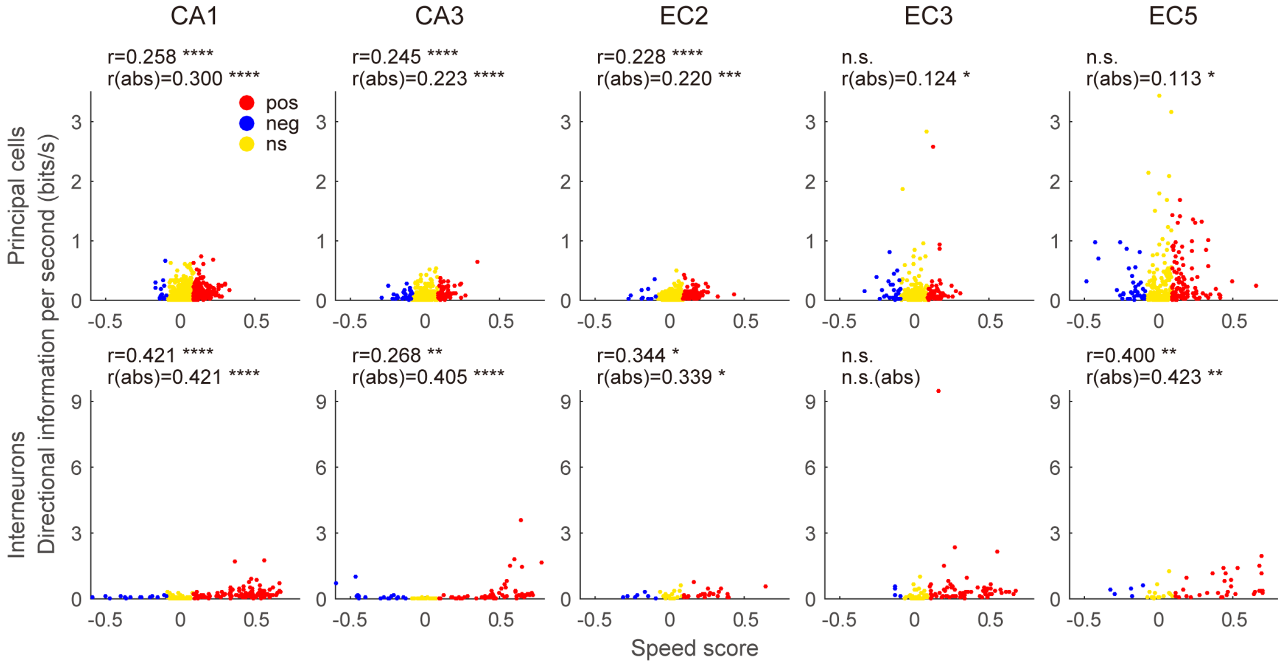

Figure S4. Relationship between directional information per second (bits/s) and speed score.

Directional information per second (bits/s) and speed score of individual neurons are shown. Principal neurons (top) and interneurons (bottom) of CA1, CA3, EC2, EC3, and EC5 are shown separately. r, correlation coefficient between directional information per second (bits/s) and speed score. r(abs), correlation coefficient between directional information per second (bits/s) and absolute value of speed score. * $P < 0.05$, ** $P < 0.01$, *** $P < 0.001$, **** $P < 0.0001$. n.s., the correlation between directional information per second (bits/s) and speed score is not significant. n.s. (abs), the correlation between directional information per second (bits/s) and absolute value of speed score is not significant. Red, p-Speed cells (pos); Blue, n-Speed cells (neg); Yellow, non-speed cells (ns). Overall, we found that directional information per second (bits/s) and (absolute value of) speed score positively correlated. The discrepancy between the relationship between directional information per spike (bits/spike) vs speed score (Fig. S3) and the relationship between directional information per second (bits/s) vs speed score (this figure) can be partly explained by the positive correlation between firing rate and (absolute value of) speed score (Fig. 2h and in the Results).

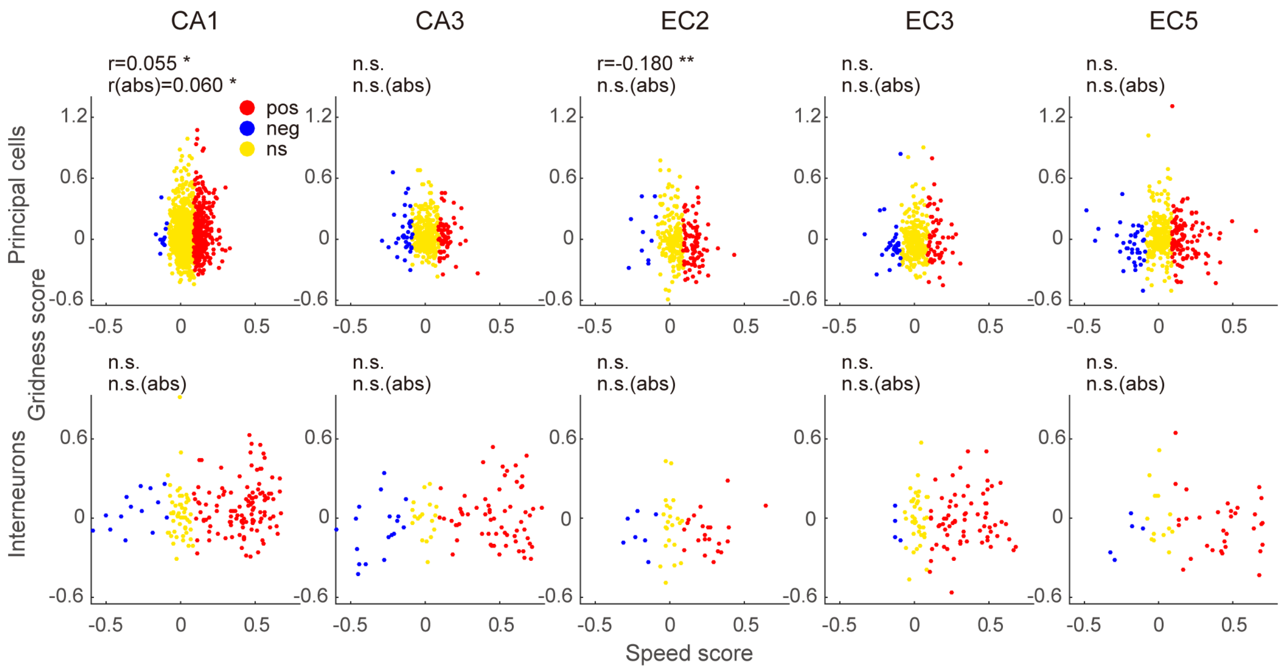

Figure S5. Relationship between gridness score and speed score.

Gridness score and speed score of individual neurons are shown. Principal neurons (top) and interneurons (bottom) of CA1, CA3, EC2, EC3, and EC5 are shown separately. r, correlation coefficient between gridness score and speed score. r(abs), correlation coefficient between gridness score and absolute value of speed score. * $P < 0.05$, ** $P < 0.01$. n.s., the correlation between gridness score and speed score is not significant. n.s. (abs), the correlation between gridness score and absolute value of speed score is not significant. Red, p-Speed cells (pos); Blue, n-Speed cells (neg); Yellow, non-speed cells (ns).

Table S2. Speed score of p-Speed cells.

Means and standard deviations of speed score are summarised.

Principal neurons and interneurons in each region (same data as in Fig. 4a,b)

Region	Principal neurons	Interneurons
CA1	0.140 ± 0.043	0.413 ± 0.156
CA3	0.140 ± 0.049	0.478 ± 0.180
EC2	0.159 ± 0.054	0.264 ± 0.125
EC3	0.149 ± 0.051	0.346 ± 0.156
EC5	0.182 ± 0.096	0.433 ± 0.200

Stellate and pyramidal cells in EC2 (same data as Fig. 7f)

Region	Stellate cells	Pyramidal cells
EC2	0.127 ± 0.035	0.182 ± 0.056

Table S3. Speed slope of p-Speed cells.

Means and standard deviations of speed slope [Hz/(cm/s)] and normalised speed slope (s/cm) are summarised.

Speed slope [Hz/(cm/s)]

Principal neurons and interneurons in each region (same data as in Fig. 4c,d)

Region	Principal neurons	Interneurons
CA1	0.050 ± 0.039	0.567 ± 0.334
CA3	0.032 ± 0.025	0.407 ± 0.268
EC2	0.050 ± 0.033	0.252 ± 0.250
EC3	0.039 ± 0.028	0.427 ± 0.294
EC5	0.055 ± 0.073	0.526 ± 0.375

Stellate and pyramidal cells in EC2 (same data as Fig. 7g)

Region	Stellate cells	Pyramidal cells
EC2	0.048 ± 0.035	0.050 ± 0.033

Normalised speed slope (s/cm)

Principal neurons and interneurons in each region (same data as in Fig. 4e,f)

Region	Principal neurons	Interneurons
CA1	0.043 ± 0.022	0.034 ± 0.017
CA3	0.054 ± 0.029	0.033 ± 0.016
EC2	0.028 ± 0.012	0.023 ± 0.016
EC3	0.028 ± 0.016	0.027 ± 0.020
EC5	0.048 ± 0.027	0.034 ± 0.019

Stellate and pyramidal cells in EC2 (same data as Fig. 7h)

Region	Stellate cells	Pyramidal cells
EC2	0.024 ± 0.009	0.030 ± 0.013

Table S4. Speed information of p-Speed cells.

Means and standard deviations of speed information per second (bits/s) and speed information per spike (bits/spike) are summarised.

Speed information per second (bits/s)

Principal neurons and interneurons in each region (same data as in Fig. 4g,h)

Region	Principal neurons	Interneurons
CA1	0.094 ± 0.077	0.806 ± 0.535
CA3	0.116 ± 0.137	0.970 ± 0.961
EC2	0.075 ± 0.070	0.254 ± 0.345
EC3	0.067 ± 0.076	0.578 ± 0.594
EC5	0.113 ± 0.187	0.920 ± 0.809

Stellate and pyramidal cells in EC2 (same data as Fig. 7i)

Region	Stellate cells	Pyramidal cells
EC2	0.068 ± 0.067	0.077 ± 0.069

Speed information per spike (bits/spike)

Principal neurons and interneurons in each region (same data as in Fig. 4i,j)

Region	Principal neurons	Interneurons
CA1	0.110 ± 0.123	0.058 ± 0.065
CA3	0.248 ± 0.331	0.082 ± 0.071
EC2	0.048 ± 0.044	0.039 ± 0.047
EC3	0.067 ± 0.091	0.045 ± 0.054
EC5	0.114 ± 0.103	0.065 ± 0.058

Stellate and pyramidal cells in EC2 (same data as Fig. 7j)

Region	Stellate cells	Pyramidal cells
EC2	0.038 ± 0.032	0.056 ± 0.051

Table S5. Preferred temporal shifts of p-Speed cells.

Means and standard deviations of preferred temporal shifts (ms) are summarised. Statistically significant retrospective (green) and prospective (red) speed representation are highlighted.

All cells (same data as Fig. 5a)

Region	Principal neurons	Interneurons
CA1	–145 ± 450	–193 ± 308
CA3	–112 ± 425	–44 ± 421
EC2	7 ± 406	343 ± 462
EC3	–254 ± 473	–50 ± 414
EC5	–121 ± 449	–20 ± 346

Theta modulated cells (same data as Fig. 5c)

Region	Principal neurons	Interneurons
CA1	–147 ± 456	–186 ± 324
CA3	–68 ± 435	–14 ± 410
EC2	173 ± 310	417 ± 375
EC3	–366 ± 531	240 ± 539
EC5	–208 ± 383	–256 ± 555

Theta non-modulated cells (same data as Fig. 5d)

Region	Principal neurons	Interneurons
CA1	–119 ± 358	–197 ± 300
CA3	–321 ± 314	–118 ± 449
EC2	–101 ± 427	92 ± 676
EC3	–212 ± 449	–123 ± 344
EC5	–106 ± 461	26 ± 285

Stellate and pyramidal cells in EC2 (same data as Fig. 7k)

Region	Stellate cells	Pyramidal cells
EC2	–87 ± 445	103 ± 354

Putative PV-expressing and SOM-expressing interneurons in CA1 and CA3 (same data as Figs. 8g,h)

Region	PV	SOM
CA1	–203 ± 302	
CA3	85 ± 305	–345 ± 451

References

1 Kropff, E., Carmichael, J. E., Moser, M. B. & Moser, E. I. Speed cells in the medial entorhinal cortex. *Nature* **523**, 419-424, doi:10.1038/nature14622 (2015).
